# The *U2AF1*^S34F^ mutation induces lineage-specific splicing alterations in myelodysplastic syndromes

**DOI:** 10.1172/JCI91363

**Published:** 2017-04-24

**Authors:** Bon Ham Yip, Violetta Steeples, Emmanouela Repapi, Richard N. Armstrong, Miriam Llorian, Swagata Roy, Jacqueline Shaw, Hamid Dolatshad, Stephen Taylor, Amit Verma, Matthias Bartenstein, Paresh Vyas, Nicholas C.P. Cross, Luca Malcovati, Mario Cazzola, Eva Hellström-Lindberg, Seishi Ogawa, Christopher W.J. Smith, Andrea Pellagatti, Jacqueline Boultwood

**Affiliations:** 1 Bloodwise Molecular Haematology Unit, Nuffield Division of Clinical Laboratory Sciences, Radcliffe Department of Medicine, University of Oxford, and BRC Blood Theme, National Institute for Health Research (NIHR) Oxford Biomedical Centre, Oxford University Hospital, Oxford, United Kingdom.; 2 The Computational Biology Research Group, Weatherall Institute of Molecular Medicine, University of Oxford, Oxford, United Kingdom.; 3 Department of Biochemistry, Downing Site, University of Cambridge, Cambridge, United Kingdom.; 4 Albert Einstein College of Medicine, Bronx, New York, USA.; 5 Medical Research Council, Molecular Hematology Unit, Weatherall Institute of Molecular Medicine, University of Oxford, and Department of Hematology, Oxford University Hospital National Health Service Trust, Oxford, United Kingdom.; 6 Faculty of Medicine, University of Southampton, Southampton, and National Genetics Reference Laboratory (Wessex), Salisbury, United Kingdom.; 7 Fondazione IRCCS Policlinico San Matteo and University of Pavia, Pavia, Italy.; 8 Center for Hematology and Regenerative Medicine, Karolinska University Hospital Huddinge, Stockholm, Sweden.; 9 Department of Pathology and Tumor Biology, Kyoto University, Kyoto, Japan.

## Abstract

Mutations of the splicing factor–encoding gene *U2AF1* are frequent in the myelodysplastic syndromes (MDS), a myeloid malignancy, and other cancers. Patients with MDS suffer from peripheral blood cytopenias, including anemia, and an increasing percentage of bone marrow myeloblasts. We studied the impact of the common *U2AF1*^S34F^ mutation on cellular function and mRNA splicing in the main cell lineages affected in MDS. We demonstrated that *U2AF1*^S34F^ expression in human hematopoietic progenitors impairs erythroid differentiation and skews granulomonocytic differentiation toward granulocytes. RNA sequencing of erythroid and granulomonocytic colonies revealed that *U2AF1*^S34F^ induced a higher number of cassette exon splicing events in granulomonocytic cells than in erythroid cells. *U2AF1*^S34F^ altered mRNA splicing of many transcripts that were expressed in both cell types in a lineage-specific manner. In hematopoietic progenitors, the introduction of isoform changes identified in the *U2AF1*^S34F^ target genes *H2AFY*, encoding an H2A histone variant, and *STRAP*, encoding serine/threonine kinase receptor–associated protein, recapitulated phenotypes associated with *U2AF1*^S34F^ expression in erythroid and granulomonocytic cells, suggesting a causal link. Furthermore, we showed that isoform modulation of *H2AFY* and *STRAP* rescues the erythroid differentiation defect in *U2AF1*^S34F^ MDS cells, suggesting that splicing modulators could be used therapeutically. These data have critical implications for understanding MDS phenotypic heterogeneity and support the development of therapies targeting splicing abnormalities.

## Introduction

The myelodysplastic syndromes (MDS) are a heterogeneous group of clonal hematopoietic stem cell malignancies characterized by ineffective hematopoiesis resulting in peripheral blood cytopenias of the myeloid lineage, including anemia and neutropenia. Patients with MDS show increasing bone marrow myeloid blasts as the disease progresses, and approximately 40% of these patients develop acute myeloid leukemia (AML). MDS is as common as de novo AML, with an incidence of 4 per 100,000 per year (1–4). Patients with the more advanced MDS subtypes (refractory anemia with excess blasts 1 and 2) have a median overall survival of less than 2 years, highlighting the severity of these diseases (1, 2, 4). The recent finding that splicing factor genes are the most commonly mutated genes found in MDS (5, 6) revealed a new leukemogenic pathway involving spliceosomal dysfunction in this disorder. Over half of all patients with MDS carry spliceosome gene mutations (6, 7), with splicing factor 3b subunit 1 (*SF3B1*), serine and arginine–rich splicing factor 2 (*SRSF2*), U2 small nuclear RNA auxiliary factor 1 (*U2AF1*), and zinc finger CCCH-type, RNA-binding motif and serine/arginine rich 2 (*ZRSR2*) being the most frequently mutated splicing factor genes (6). The common spliceosome mutations in MDS have differing prognostic impacts (8–10) and to some extent define distinct clinical phenotypes (5, 8, 11). While at present there is a lack of extensive direct evidence that the alterations in pre-mRNA splicing caused by mutations in splicing factors are the main mechanism driving the disease in MDS, aberrant splicing of some key downstream target genes (e.g., ATP-binding cassette subfamily B member 7 [*ABCB7*] and enhancer of zeste 2 polycomb repressive complex 2 subunit [*EZH2*]) linked to splicing factor gene (*SF3B1* and *SRSF2*) mutations has been shown to be associated with certain MDS disease aspects and phenotypes (12, 13).

Pre-mRNA splicing involves the excision of intronic sequences from pre-mRNAs (14) and is performed by the spliceosome, a complex of 5 small nuclear ribonucleoproteins (snRNPs) and other supplementary proteins. U2AF1 (also known as U2AF35) is a U2 auxiliary factor that forms a heterodimer with U2AF2 (also known as U2AF65) for the recognition of the 3′ splice site and subsequent recruitment of U2 snRNPs during pre-mRNA splicing (15, 16). Mutations in *U2AF1* have been found in approximately 11% of patients with MDS (6, 17), making *U2AF1* one of the most commonly mutated genes in this disease. Mutations in *U2AF1* also occur in the closely related condition AML at a frequency of approximately 4% (18), as well as in lung adenocarcinoma and other cancers (18, 19). *U2AF1* mutations are associated with a worse overall survival in MDS patients and a higher risk of transformation to AML (11, 20, 21). *U2AF1* mutations almost exclusively occur in 2 highly conserved amino acid positions, S34 and Q157, within the 2 zinc finger domains of the protein (6). There is clear evidence in yeast showing that the zinc finger domains in U2AF1 recognize RNA (6). The high percentage of sequence identity in the zinc finger domains between yeast and human U2AF1 (6) suggests that the zinc finger domains in human U2AF1 are also RNA binding (22). The presence of missense mutational hotspots and the absence of nonsense/frameshift mutations suggest that *U2AF1* mutations are gain-of-function or change-of-function/neomorphic mutations (6).

Abnormal RNA splicing, with cassette exon splicing being the most frequent type of event, has been reported in bone marrow samples from *U2AF1*-mutant MDS and AML patients (18, 23, 24). Several studies demonstrated that differentially spliced exons exhibited different consensus nucleotides at the –3 and +1 positions flanking the AG dinucleotide of the 3′ splice site (18, 23–25). Thymidine (uridine) was observed less frequently than cytosine at the –3 position of the 3′ splice site in the *U2AF1* S34F mutant compared with *U2AF1* WT samples (18, 23, 25).

Recently, Shirai et al. generated a doxycycline-inducible transgenic mouse model of *U2AF1* S34F mutation displaying some phenotypes that are closely associated with MDS (26). This transgenic murine model sheds light on the role of this mutation in altering hematopoesis and pre-mRNA splicing in the mouse (26). An investigation of the lineage-specific effect of *U2AF1* S34F mutation on human hematopoiesis could provide new insights into the molecular pathogenesis of *U2AF1*-mutant MDS and illuminate how this mutation impacts the MDS phenotype.

Here, we demonstrate that the *U2AF1* S34F mutation exhibits lineage specificity in altering pre-mRNA splicing of downstream target genes, resulting in different phenotypes in the different myeloid lineages that are involved in MDS.

## Results

### Expression of U2AF1^S34F^ in hematopoietic progenitors.

**Figure 1:**
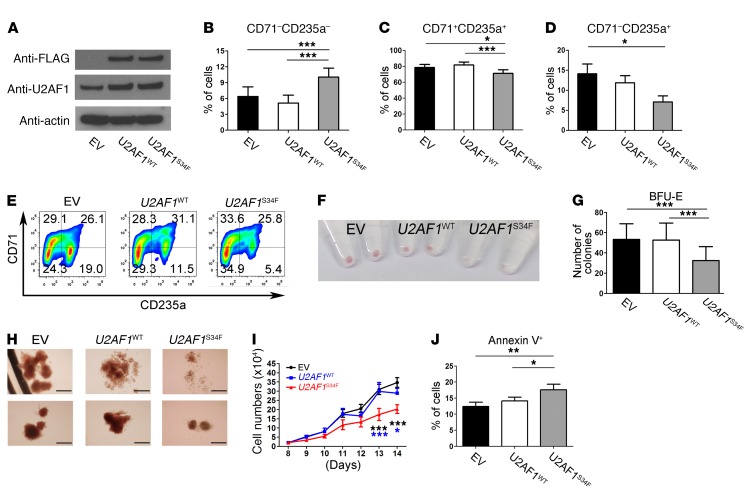
Expression of U2AF1^S34F^ impairs erythroid differentiation. (**A**) Western blots showing expression levels of U2AF1^S34F^ and U2AF1^WT^ protein in transduced erythroid cells harvested on day 11. An anti-U2AF1 antibody was used to measure total U2AF1 protein, while an anti-FLAG antibody was used to measure the exogenous U2AF1^S34F^ or U2AF1^WT^ protein produced by the vector. (**B**–**D**) Erythroid differentiation was measured by flow cytometry using expression of CD71 and CD235a cell-surface markers. (**B**) Nonerythroid (CD71^–^CD235a^–^) and (**C**) intermediate erythroid (CD71^+^CD235a^+^) cell populations on day 11 of culture and (**D**) late erythroid (CD71^–^CD235a^+^) cell population on day 14 of culture. (**E**) Representative flow cytometric plots showing impaired erythroid differentiation on day 14 (*n* = 8). (**F**) Image of erythroid cell pellets on day 14 of culture for visual determination of hemoglobinization (*n* = 8). (**G**) Number of BFU-E colonies obtained from hematopoietic CD34^+^ progenitors transduced with EV, *U2AF1*^WT^, or *U2AF1*^S34F^ after 14 days in methylcellulose (colony-forming cell assays). (**H**) Representative images of BFU-E colonies produced from hematopoietic CD34^+^ progenitors transduced with EV, *U2AF1*^WT^, or *U2AF1*^S34F^, respectively (*n* = 7). Scale bars: 100 μm. (**I**) Cell counts for *U2AF1*^S34F^ erythroid cells from day 8 to day 14 of culture compared with counts for EV and *U2AF1*^WT^ controls. (**J**) Apoptosis as measured by annexin V staining and flow cytometry in erythroblasts harvested on day 11 of culture. Results shown in panels **B**–**D** were obtained from 8 independent experiments, those shown in panels **G** and **I** were obtained from 7 independent experiments, and results shown in panel **J** were obtained from 6 independent experiments. Data represent the mean ± SEM. *P* values in panels **B**–**D**, **G**, and **J** were calculated by repeated-measures 1-way ANOVA with Tukey’s post-hoc test. *P* values in panel **I** were calculated by 2-way ANOVA with Bonferroni’s post test. *P < 0.05, **P < 0.01, and ***P < 0.001.

**Figure 2:**
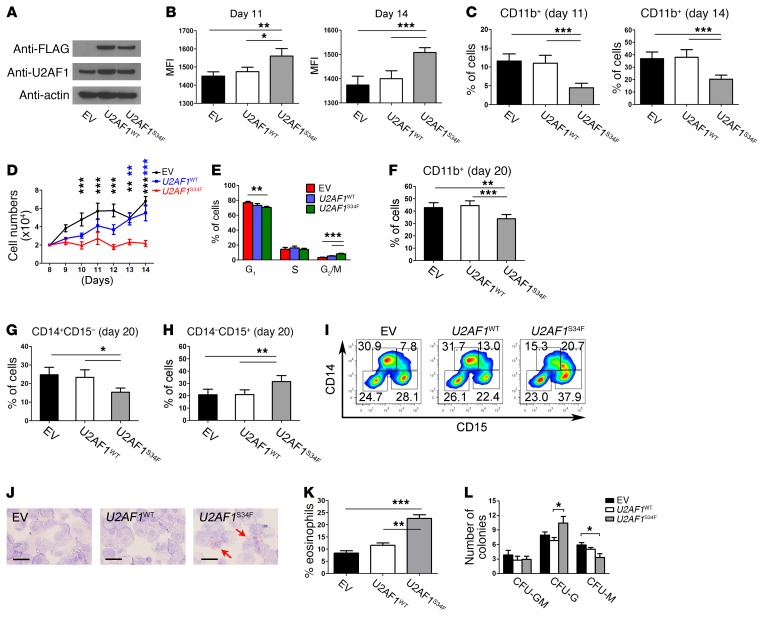
Expression of U2AF1^S34F^ skews granulomonocytic differentiation toward granulocytes. (**A**) Expression levels of U2AF1^S34F^ and U2AF1^WT^ protein in transduced granulomonocytic cells on day 11. Anti-U2AF1 and anti-FLAG antibodies were used to measure total U2AF1 protein and exogenous U2AF1^S34F^ or U2AF1^WT^ protein produced by the vector, respectively. (**B**) Median fluorescence intensity (MFI) of forward scatter (an indication of cell size) of granulomonocytic cells on days 11 and 14. (**C**) Percentage of CD11b^+^ cells in granulomonocytic cultures on days 11 and 14. (**D**) Cell counts for *U2AF1*^S34F^ granulomonocytic cells from day 8 (the day when Geneticin selection was complete) to day 14 compared with EV and *U2AF1*^WT^ controls. (**E**) Cell-cycle analysis of granulomonocytic cells on day 11. (**F**) Percentage of CD11b^+^ cells in granulomonocytic cultures on day 20. (**G** and **H**) Percentages of (**G**) CD14^+^CD15^–^ monocytic cells and (**H**) CD14^–^CD15^+^ granulocytic cells in granulomonocytic cultures on day 20. (**I**) Representative flow cytometric plots on day 20 (*n* = 7). (**J**) Representative images of May-Grünwald-Giemsa–stained granulomonocytic cells on day 20 (*n* = 7). The red arrows indicate eosinophils. Scale bars: 25 μm. (**K**) Percentage of eosinophils per 100 cells on day 20. (**L**) Number of CFU granulocytes-macrophages (CFU-GM), CFU granulocytes (CFU-G), and CFU macrophages (CFU-M) obtained from hematopoietic CD34^+^ progenitors transduced with EV, *U2AF1*^WT^, or *U2AF1*^S34F^ after 14 days in methylcellulose. Results shown in panels **B**–**H**, **K**, and **L** were obtained from 6, 8, 7, 6, 7, 7, 7, 6, and 7 independent experiments, respectively. Data represent the mean ± SEM. *P* values in panels **B**, **C**, **E**–**H**, **K** and **L** were calculated by repeated-measures 1-way ANOVA with Tukey’s post-hoc test. *P* values in panel **D** were calculated by 2-way ANOVA with Bonferroni’s post test. *P < 0.05, **P < 0.01, and ***P < 0.001.

To study the impact of the *U2AF1* S34F mutation on erythroid and granulomonocytic differentiation, we first overexpressed the *U2AF1* S34F mutant (*U2AF1*^S34F^) and *U2AF1* WT (*U2AF1*^WT^) in primary human bone marrow CD34^+^ progenitor cells by retroviral transduction. Transduced progenitor cells were then cultured under erythroid or granulomonocytic conditions for differentiation into erythroid and granulomonocytic cells, respectively (Supplemental Figure 1A; supplemental material available online with this article; https://doi.org/10.1172/JCI91363DS1). The gene expression levels of *U2AF1*^S34F^ and *U2AF1*^WT^ compared with levels in the empty vector (EV) control were confirmed by real-time quantitative PCR (qRT-PCR) in transduced cells harvested on day 11 (Supplemental Figure 1B). The expression of *U2AF1*^S34F^ in transduced cells was confirmed by Sanger sequencing (Supplemental Figure 1C). Expression of U2AF1^S34F^ and U2AF1^WT^ protein in transduced cells harvested on day 11 was detected using anti-FLAG and anti-U2AF1 antibodies (Figure 1A and Figure 2A). Overexpression of exogenous U2AF1^S34F^ and U2AF1^WT^ protein, driven by retroviral vectors (anti-FLAG antibody), resulted in a modest increase (approximately 1.5- to 2.0-fold increase, i.e., not a large excess compared with the levels observed in the EV control) of total U2AF1 protein levels (anti-U2AF1 antibody) in *U2AF1*^S34F^- and *U2AF1*^WT^-transduced cells throughout erythroid and granulomonocytic differentiation (Supplemental Figure 1D).

### U2AF1^S34F^ impairs erythroid differentiation.

To investigate the effect of *U2AF1*^S34F^ on erythroid differentiation, transduced hematopoietic progenitors were cultured using a method developed to study the generation of erythroblasts (27), and erythroblasts were harvested on day 11 and day 14 of culture for flow cytometric measurement of the erythroid cell-surface markers CD71 and CD235a (Figure 1, B–E). We observed a significant increase in the CD71^–^CD235a^–^ nonerythroid cell population (Figure 1B) and a significant decrease in the CD71^+^CD235a^+^ intermediate erythroid cell population (Figure 1C) in *U2AF1*^S34F^ erythroblasts on day 11 compared with the *U2AF1*^WT^ and EV controls. We observed a decrease in the late CD71^–^CD235a^+^ erythroid cell population in *U2AF1*^S34F^ erythroblasts on day 14 compared with the *U2AF1*^WT^ and EV controls (Figure 1, D and E). Examination of erythroblasts on day 14 revealed that *U2AF1*^S34F^ erythroblasts had defective hemoglobinization compared with the *U2AF1*^WT^ and EV controls (Figure 1F). To further characterize the effect of *U2AF1*^S34F^ on erythroid differentiation, we performed colony-forming cell assays and found that *U2AF1*^S34F^-transduced progenitors produced a significantly lower number of burst-forming unit–erythroid (BFU-E) colonies than did the *U2AF1*^WT^ and EV controls after 14 days in culture (Figure 1G). Morphological examination of BFU-E colonies revealed that *U2AF1*^S34F^ inhibited colony growth, resulting in smaller colonies, and impaired hemoglobinization (Figure 1H). Transduced erythroblasts underwent Geneticin selection on day 3 following retroviral transduction. The cells were harvested on day 8 for cell growth assays until day 14 in culture (Figure 1I). *U2AF1*^S34F^ erythroblasts showed impaired cell growth compared with the *U2AF1*^WT^ and EV controls (Figure 1I). We found no change in cell-cycle pattern among the samples (Supplemental Figure 1E), but observed a significant increase in apoptosis in *U2AF1*^S34F^ erythroblasts harvested on day 11 compared with the *U2AF1*^WT^ and EV controls (Figure 1J). These results indicate that *U2AF1*^S34F^ impaired cell growth and increased apoptosis in erythroblasts and led to impaired differentiation.

### U2AF1^S34F^ skews granulomonocytic differentiation toward granulocytes.

To investigate the effects of *U2AF1*^S34F^ on granulomonocytic differentiation, transduced hematopoietic progenitors were cultured under granulomonocytic differentiating conditions (28), and granulomonocytic cells were harvested on day 11 and day 14 of culture for flow cytometric measurement of the myeloid cell-surface markers CD11b, CD14, and CD15. CD11b is a myeloid cell-surface marker expressed on granulocytes, monocytes, and macrophages (29). CD14 and CD15 are expressed predominantly on monocytes and granulocytes, respectively, and were used as lineage discriminators between monocytic and granulocytic cell populations (30). Given the signal intensity of forward scatter (as a measure of cell size), *U2AF1*^S34F^ granulomonocytic cells were larger than the *U2AF1*^WT^ and EV controls (Figure 2B and Supplemental Figure 1F). We observed a significant decrease in the CD11b^+^ cell population in *U2AF1*^S34F^ granulomonocytic cells compared with that seen in the *U2AF1*^WT^ and EV controls on days 11 and 14 (Figure 2C). However, no difference in CD14^+^ or CD15^+^ cell populations was observed among samples at these 2 time points (data not shown). *U2AF1*^S34F^ granulomonocytic cells showed impaired cell growth compared with the *U2AF1*^WT^ and EV controls (Figure 2D). We observed no difference in apoptosis in *U2AF1*^S34F^ granulomonocytic cells (Supplemental Figure 1G), however, *U2AF1*^S34F^ triggered a G_2_/M cell-cycle arrest in granulomonocytic cells compared with the *U2AF1*^WT^ and EV controls (Figure 2E). These data demonstrate impaired growth and differentiation of *U2AF1*^S34F^ granulomonocytic cells.

We also evaluated granulomonocytic differentiation on day 20 of culture and observed a significant decrease in the CD11b^+^ cell population in *U2AF1*^S34F^ granulomonocytic cells compared with *U2AF1*^WT^ and EV controls (Figure 2F). Moreover, we observed a significant decrease in the CD14^+^ monocytic cell population and a concomitant significant increase in the CD15^+^ granulocytic cell population in *U2AF1*^S34F^ granulomonocytic cells compared with the *U2AF1*^WT^ and EV controls, indicating a skewing effect of *U2AF1*^S34F^ on granulomonocytic differentiation (Figure 2, G–I). To confirm this phenotype, granulomonocytic cells were stained with May-Grünwald and Giemsa for morphological examination (Figure 2, J and K). Consistent with the flow cytometric data (Figure 2, G –I), we observed an expansion of the granulocyte population, specifically eosinophils, in *U2AF1*^S34F^ granulomonocytic cells compared with *U2AF1*^WT^ and EV controls (Figure 2, J and K). Furthermore, *U2AF1*^S34F^-transduced progenitors produced a significantly lower number of CFU-M colonies and a significantly higher number of CFU-G colonies in myeloid colony–forming cell assays (Figure 2L). Our results indicate that *U2AF1*^S34F^ perturbs granulomonocytic cells by skewing their differentiation from monocytes toward granulocytes.

### U2AF1^S34F^ differentially alters splicing in erythroid and granulomonocytic colonies.

**Figure 3:**
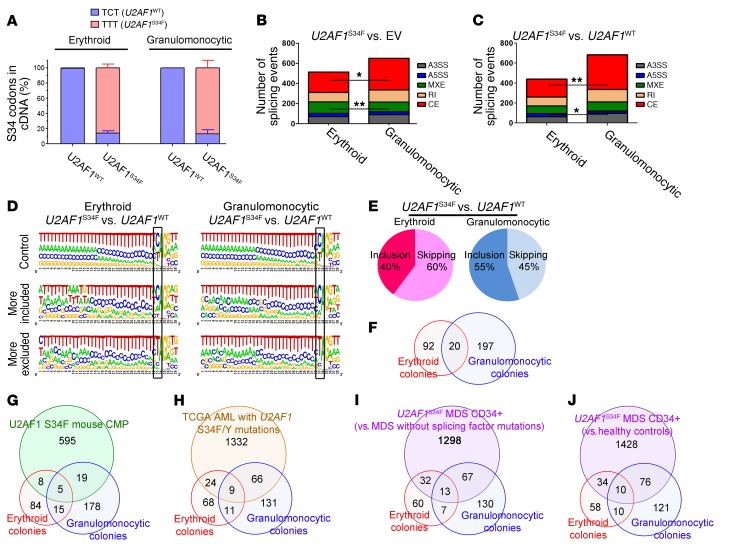
*U2AF1*^S34F^ differentially alters splicing of target genes in erythroid and granulomonocytic colonies. (**A**) Quantification of *U2AF1* WT (TCT) and S34F mutant (TTT) mRNA in erythroid and granulomonocytic colonies, determined by pyrosequencing. (**B** and **C**) Aberrant splicing events associated with *U2AF1*^S34F^, including breakdown by event type, in erythroid and granulomonocytic colonies for (**B**) *U2AF1*^S34F^ versus EV and (**C**) *U2AF1*^S34F^ versus *U2AF1*^WT^. (**D**) Sequence logos for 3′ splice sites of cassette exons that were unaffected (top row), more included (middle row), or more skipped (bottom row) in response to *U2AF1*^S34F^ compared with *U2AF1*^WT^. (**E**) Distribution of exon inclusion and skipping events within the total number of regulated cassette exon events in the comparison of *U2AF1*^S34F^ versus *U2AF1*^WT^ in erythroid and granulomonocytic colonies. (**F**) Venn diagram showing the overlap among the genes that contained aberrant splicing events induced by *U2AF1*^S34F^ in erythroid and granulomonocytic colonies in our study. (**G**–**J**) Venn diagrams showing the overlap among the genes that contained aberrant splicing events induced by *U2AF1*^S34F^ in different RNA-seq data sets: (**G**) transgenic mouse CMPs expressing U2AF1^S34F^ and erythroid colonies and granulomonocytic colonies in our study; (**H**) TCGA AML patient samples with *U2AF1* S34 mutations and erythroid colonies and granulomonocytic colonies in our study; (**I**) *U2AF1*^S34F^ MDS CD34^+^ bone marrow cells (versus MDS cases without splicing factor gene mutations) and erythroid colonies and granulomonocytic colonies in our study; and (**J**) *U2AF1*^S34F^ MDS CD34^+^ bone marrow cells (versus healthy controls) and erythroid colonies and granulomonocytic colonies in our study. Results in panel **A** are shown as the mean ± SEM and were obtained from 3 independent experiments. *P* values in panel **B** and **C** were calculated by Fisher’s exact test with Bonferroni’s correction. *P < 0.05 and **P < 0.01. A3SS, alternative 3′ splice site; A5SS, alternative 5′ splice site; MXE, mutually exclusive exon; RI, retained intron; SE, skipped (cassette) exon.

In order to investigate the effects of the presence of *U2AF1*^S34F^ on pre-mRNA splicing in cells committed toward the myeloid or erythroid lineage, we performed RNA sequencing (RNA-seq) on individual erythroid and granulomonocytic colonies formed in colony-forming cell assays by bone marrow CD34^+^ cells transduced with *U2AF1*^S34F^, *U2AF1*^WT^, or EV control. The *U2AF1*^S34F^ variant allele frequency was greater than 80% in erythroid and granulomonocytic colonies expressing the *U2AF1* S34F mutation, as measured by pyrosequencing (Figure 3A). Replicate MATS (rMATS), a computational tool designed for the detection of differential alternative splicing from replicate RNA-seq data (31), was used for RNA-seq data analysis. A total of 506 splicing events (347 genes) and 439 splicing events (300 genes) were identified in *U2AF1*^S34F^ erythroid colonies compared with the *U2AF1*^WT^ and EV controls, respectively (see Supplemental data file 1). A total of 643 splicing events (447 genes) and 676 splicing events (474 genes) were identified in *U2AF1*^S34F^ granulomonocytic colonies compared with the *U2AF1*^WT^ and EV controls, respectively (see Supplemental data file 1). Alteration in cassette exon splicing (i.e., exons that are either included or spliced out, showing increased or decreased inclusion levels, respectively) was the most common type of aberrant splicing event induced by *U2AF1*^S34F^ in both erythroid and granulomonocytic colonies (Figure 3, B and C). We observed a significantly higher number of regulated cassette exon events in granulomonocytic *U2AF1*^S34F^ colonies compared with erythroid *U2AF1*^S34F^ colonies in both comparisons with the *U2AF1*^WT^ and EV controls (Figure 3, B and C). This increase in the number of cassette exon events accounts for the higher number of total aberrant splicing events associated with granulomonocytic *U2AF1*^S34F^ colonies compared with erythroid *U2AF1*^S34F^ colonies (Figure 3, B and C).

We investigated the properties of misregulated cassette exons and cassette exons that were unaffected by *U2AF1*^S34F^ compared with *U2AF1*^WT^ (Figure 3D and Supplemental Figure 2). Cassette exons that were more included or that were unregulated showed the normal preference for CAG 3′ splice sites. In contrast, exons that were more skipped in response to *U2AF1*^S34F^ showed a strong enrichment for TAG 3′ splice sites (Figure 3D, position 33 on the sequence logos), as observed previously (18, 23, 24, 26). These exons also had significantly weaker 5′ splice sites than did the *U2AF1*^S34F^ unregulated exons (Supplemental Figure 2). Their 3′ splice sites were also weaker than those in unregulated exons (erythroid only), while their branch point strengths did not differ significantly. While we could readily detect differences between exons that were more included or skipped in response to *U2AF1*^S34F^, we observed no differences between the properties of exons regulated in erythroid or granolumonocytic cells. However, we found that the majority of regulated cassette exons (*U2AF1*^S34F^ vs. *U2AF1*^WT^) were more skipped in erythroid cells (60% more skipped), while in granulomonocytic cells only 45% were more skipped (Figure 3E).

The number of genes showing significant aberrant splicing events in both comparisons of *U2AF1*^S34F^ with EV and *U2AF1*^S34F^ with *U2AF1*^WT^ were 112 in erythroid colonies and 217 in granulomonocytic colonies (Figure 3F and Supplemental data file 2). A total of 92 genes showed aberrant splicing events in erythroid colonies only and 197 genes in granulomonocytic colonies only (Figure 3F and Supplemental data file 2). The large majority (≥95%) of these genes that were aberrantly spliced in either the erythroid or granulomonocytic lineage only were also expressed in the other lineage (Supplemental Figure 3A). Twenty genes were common in the lists of aberrantly spliced genes in erythroid and granulomonocytic *U2AF1*^S34F^ colonies (Figure 3F and Supplemental data file 2). For the genes that were differentially spliced in the erythroid lineage, we found that the distribution of their expression levels (log_2_ reads per kilobase per million [RPKM]) was comparable in the erythroid and granulomonocytic lineages. Likewise, for the genes that were differentially spliced in the granulomonocytic lineage, we found that the distribution of their expression levels (log_2_ RPKM) was comparable in the erythroid and granulomonocytic lineages (Supplemental Figure 3B). The limited overlap of aberrantly spliced genes between erythroid and granulomonocytic colonies suggests that the splicing of different sets of genes was altered in the erythroid and granulomonocytic lineages. We performed gene ontology analysis on the lists of significant genes showing aberrant splicing events identified by the rMATS pipeline using GOseq (Bioconductor; http://bioconductor.org/packages/release/bioc/html/goseq.html). The significant main ontology themes for the comparison of erythroid *U2AF1*^S34F^ colonies with EV and/or *U2AF1*^WT^ are related to heme processing and mRNA processing (see Supplemental data files 3 and 4). Taken together, these data indicate that *U2AF1*^S34F^ alters target genes in a lineage-specific manner, driving different phenotypes in different myeloid lineages.

To identify common aberrantly spliced genes associated with *U2AF1* mutations, we performed a comparison of our RNA-seq data on *U2AF1*^S34F^ erythroid and granulomonocytic cells with RNA-seq data from other studies, including common myeloid progenitors (CMPs) from a *U2AF1*^S34F^-transgenic mouse (26) and AML patient samples with *U2AF1* S34 mutations (23) (Figure 3, G and H, and Supplemental data files 5 and 6). Approximately 10% and 30% of the aberrantly spliced genes in our RNA-seq data set on erythroid and granulomonocytic colonies were also present in the studies of mouse CMPs and AML patient samples, respectively (Figure 3, G and H, and Supplemental data files 5 and 6). The genes that are shared across data sets represent important targets of U2AF1^S34F^.

Moreover, we performed RNA-seq on bone marrow CD34^+^ cells from 2 patients with MDS with the *U2AF1*^S34F^ mutation, 4 patients with MDS without known mutations in splicing factor genes, and 5 healthy controls. We compared the lists of aberrantly spliced genes identified by rMATS in the comparison of *U2AF1*^S34F^ MDS cases versus MDS cases without splicing factor gene mutations and versus healthy controls, with the lists of aberrantly spliced genes we identified in *U2AF1*^S34F^-transduced erythroid and granulomonocytic colonies. We found that approximately 40% of the aberrantly spliced genes identified in *U2AF1*^S34F^-transduced erythroid and granulomonocytic colonies, respectively, were also present in the lists of aberrantly spliced genes in the comparisons of *U2AF1*^S34F^ MDS cases versus MDS cases without splicing factor gene mutations and versus healthy controls (Figure 3, I and J, and Supplemental data files 1, 7, and 8).

The differences in the number of differentially spliced target genes identified in *U2AF1*^S34F^-transduced erythroid and granulomonocytic colonies in our study and the number of differentially spliced target genes identified in the other data sets mentioned above are likely due to differences in the RNA-seq analysis pipeline and/or filtering cutoff values as well as the cell type analyzed.

### Confirmation of splicing alterations in U2AF1^S34F^ erythroid and granulomonocytic cells.

We selected several aberrantly spliced genes in *U2AF1*^S34F^ erythroid and granulomonocytic colonies for confirmation of the splicing abnormality identified using rMATS. The genes were chosen on the basis of the following criteria: abnormal splicing identified in our study and also in the *U2AF1*^S34F^-transgenic mouse CMPs and/or The Cancer Genome Atlas (TCGA) AML patient samples with *U2AF1* S34 mutations (18, 23, 26); known biological function (particularly regarding hematopoiesis); and previously described involvement in tumorigenesis. We selected the H2A histone family member Y (*H2AFY*); serine/threonine kinase receptor–associated protein (*STRAP*); SWI/SNF-related, matrix associated, actin-dependent regulator of chromatin, subfamily a, member 5 (*SMARCA5*); integrin subunit β 3–binding protein (*ITGB3BP*); and ATR serine/threonine kinase (*ATR*) genes for confirmation of the mutant U2AF1-induced splice isoform changes identified by RNA-seq in these 5 genes using qRT-PCR and RT-PCR and gel electrophoresis.

**Figure 4:**
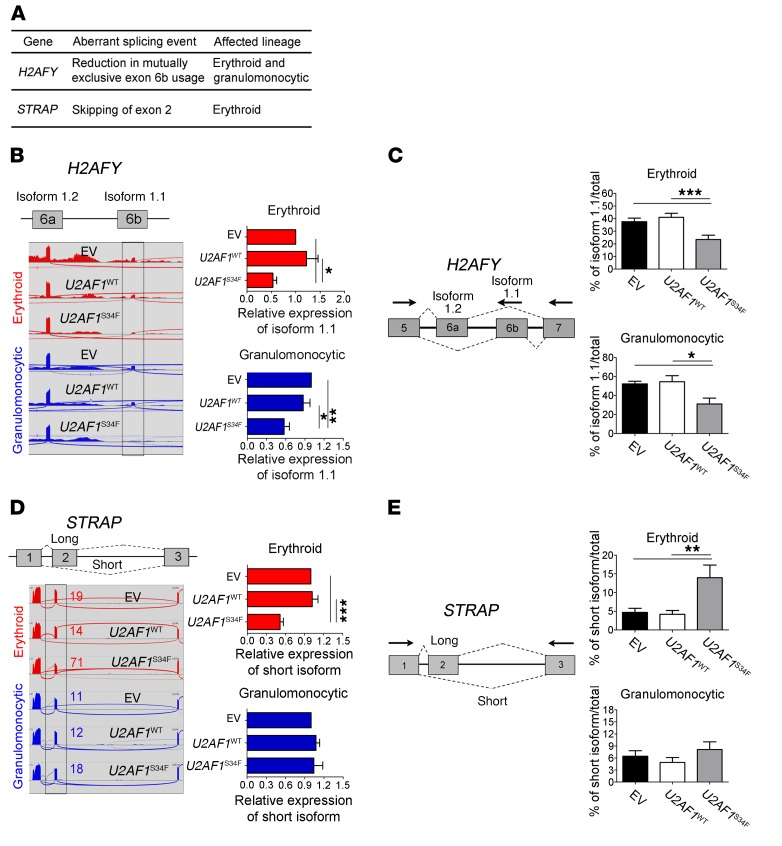
Confirmation of lineage-specific splicing alterations in *U2AF1*^S34F^ erythroid and granulomonocytic cells. (**A**) Genes of interest (*H2AFY* and *STRAP*) that exhibited differential aberrant splicing between *U2AF1*^S34F^ erythroid and granulomonocytic colonies. (**B** and **C**) Mutually exclusive exons in *H2AFY* measured by (**B**) isoform-specific qRT-PCR and confirmed by (**C**) RT-PCR and gel electrophoresis. (**D** and **E**) Exon skipping in *STRAP* measured by (**D**) isoform-specific qRT-PCR and confirmed by (**E**) RT-PCR and gel electrophoresis. In panels **B** and **D**, sashimi plots illustrate RNA-seq results for *H2AFY* and *STRAP* in erythroid and granulomonocytic colonies. For each gene, the region affected by aberrant splicing is shown, and the aberrant splicing event is highlighted in gray. In panel **D**, the qRT-PCR was specific for the long STRAP isoform, as it was not possible to design a qRT-PCR specific for the short isoform (as there are no unique exons that are specific for the short isoform). The decrease in expression levels of the long STRAP isoform observed in U2AF1^S34F^ erythroid cells is due to the aberrant splicing, which removes exon 2 from the long isoform, resulting in the generation of the short isoform and a concomitant depletion of the long isoform. Expression of the isoform associated with aberrant splicing by *U2AF1*^S34F^ in transduced cells was measured by isoform-specific qRT-PCR relative to *U2AF1*^WT^ and EV controls (red bars: erythroid cells; blue bars: granulomonocytic cells). In panels **C** and **E**, quantification of altered splicing events in gel was performed using ImageJ. Results for each bar graph were obtained from 5 independent experiments in panels **B**–**E**. Data represent the mean ± SEM. *P* values in panels **B**–**E** were calculated by repeated-measures 1-way ANOVA with Tukey’s post-hoc test. *P < 0.05 and **P < 0.01.

We identified a mutually exclusive exon splicing alteration in *H2AFY* in both *U2AF1*^S34F^ erythroid and granulomonocytic colonies (Figure 4, A–C). Decreased usage of exon 6b (which is mutually exclusive to exon 6a) in the *H2AFY* gene was observed in both *U2AF1*^S34F^ erythroid and granulomonocytic colonies compared with the corresponding *U2AF1*^WT^ and EV controls (Figure 4, B and C). We observed aberrant splicing of *STRAP* and *SMARCA5* in cells of the erythroid lineage only (Figure 4, A, D, and E, and Supplemental Figure 4, A and C), while splicing alterations of *ITGB3BP* and *ATR* were found only in granulomonocytic cells (Supplemental Figure 4, A, B, and D). Increased skipping of exon 2 of *STRAP* (Figure 4, D and E) and skipping of exon 14 of *SMARCA5* (Supplemental Figure 4C) in *U2AF1*^S34F^ cells compared with the *U2AF1*^WT^ and EV controls occurred preferentially in erythroid cells. In contrast, inclusion of exon 2 of *ITGB3BP* and inclusion of exon 47 of *ATR* were associated with *U2AF1*^S34F^ granulomonocytic cells (Supplemental Figure 4, B and D). To confirm these splicing alterations, we performed isoform-specific qRT-PCR as well as RT-PCR and gel electrophoresis to measure the isoform changes associated with U2AF1^S34F^ in transduced erythroid and granulomonocytic cells, and all splicing alterations were concordant with the RNA-seq data (Figure 4, B and D, and Supplemental Figure 4, B–D). Moreover, the full-length isoforms and the aberrant splice junctions of the selected genes (*H2AFY* and *STRAP*) were all confirmed by Sanger sequencing (Supplemental Figures 5 and 6). Our data show that U2AF1^S34F^ differentially alters splicing of target genes in a lineage-specifc manner in the erythroid and granulomonocytic lineages, supporting the hypothesis that the same splicing factor gene mutation can drive aberrant splicing of distinct genes in different cell populations.

Park et al. recently reported selection of a distal cleavage and polyadenylation (CP) site in the autophagy-related factor 7 (*Atg7*) pre-mRNA in association with the presence of the *U2AF1*^S34F^ mutation (32). This results in a decrease in ATG7 levels that leads to defective autophagy, making cells more prone to secondary mutations (32). We performed qRT-PCR to evaluate selection of the distal CP site of *ATG7* (32) in *U2AF1*^S34F^ erythroid and granulomonocytic colonies in our study. We did not observe increased usage of the distal CP site of *ATG7* in *U2AF1*^S34F^ granulomonocytic cells (distal/proximal CP site usage ratios of 1.03 and 0.98 in *U2AF1*^S34F^ and *U2AF1*^WT^, respectively, compared with the EV control), however this represents a different cell population from that studied by Park et al. (32). The expression of *ATG7* was too low for assessment in *U2AF1*^S34F^ erythroid cells.

### Functional effects of splicing aberrations associated with U2AF1^S34F^.

We performed functional studies to determine the impact of the splicing abnormalities identified in *H2AFY*, *STRAP*, and *ITGB3BP* on human erythroid and/or granulomonocytic cell growth and differentiation.

**Figure 5:**
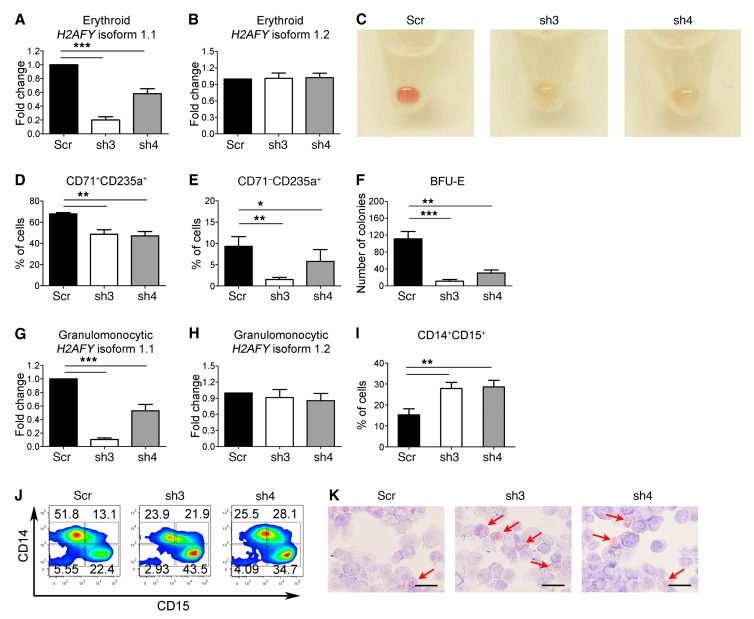
Knockdown of *H2AFY* isoform 1.1 perturbs erythroid and granulomonocytic differentiation. (**A** and **B**) Expression levels of *H2AFY* (**A**) isoform 1.1 and (**B**) isoform 1.2 were determined using isoform-specific qRT-PCR in erythroid cells with *H2AFY* isoform 1.1 knockdown. (**C**) Images of erythroid cell pellets on day 14 of culture for visual determination of hemoglobinization (*n* = 6). (**D** and **E**) Erythroid differentiation measured by expression of CD71 and CD235a cell-surface markers using flow cytometry. (**D**) Intermediate erythroid (CD71^+^CD235a^+^) cell population on day 11 of culture and (**E**) late erythroid (CD71^–^CD235a^+^) cell population on day 14 of culture. (**F**) Number of BFU-E obtained from hematopoietic CD34^+^ progenitors with *H2AFY* isoform 1.1 knockdown after 14 days in methylcellulose (colony-forming cell assays). (**G** and **H**) Expression levels of *H2AFY* (**G**) isoform 1.1 and (**H**) isoform 1.2 determined using isoform-specific qRT-PCR in granulomonocytic cells with *H2AFY* isoform 1.1 knockdown. (**I**) Percentage of CD14^+^CD15^+^ cells in granulomonocytic cultures on day 20 of culture. (**J**) Representative flow cytometric contour plots showing expression of CD14 and CD15 on day 20 of culture (*n* = 8). (**K**) Representative images of May-Grünwald-Giemsa–stained granulomonocytic cells on day 20 of culture (*n* = 8). The red arrows indicate eosinophils. Scale bars: 25 μm. Results in each bar graph were obtained from 6 independent experiments for panels **A**, **B**, and **D**–**H**, and from 8 independent experiments for panel **I**. Data represent the mean ± SEM. *P* values in panels **A**, **B**, and **D**–**I** were calculated by repeated-measures 1-way ANOVA with Tukey’s post-hoc test. *P < 0.05, **P < 0.01, and ***P < 0.001. Scr, scramble; sh3, shRNA 3; sh4, shRNA 4.

Usage of the mutually exclusive exons 6a and 6b in the *H2AFY* gene gives rise to the 2 transcript isoforms 1.2 and 1.1, respectively. Our RNA-seq data showed that *U2AF1*^S34F^ was associated with altered pre-mRNA splicing of *H2AFY,* with decreased usage of exon 6b resulting in a decrease in the expression levels of the isoform 1.1 of this gene in both erythroid and granulomonocytic colonies. To examine the effects of reduced expression of the *H2AFY* isoform 1.1 on human hematopoiesis, we designed shRNAs to specifically knock down the *H2AFY* isoform 1.1 (Figure 5, A and G), without affecting the expression of the *H2AFY* isoform 1.2 (Figure 5, B and H) in bone marrow CD34^+^ progenitor cells. Transduced progenitor cells were cultured under erythroid and granulomonocytic conditions as previously described (27, 28). Erythroblasts with *H2AFY* isoform 1.1 knockdown showed increased apoptosis (Supplemental Figure 7A) and G_1_ cell-cycle arrest (Supplemental Figure 7B). Similar to erythroblasts expressing *U2AF1*^S34F^, erythroid cells with *H2AFY* isoform 1.1 knockdown showed defective hemoglobinization on day 14 of culture compared with the scramble shRNA control (Figure 5C). Furthermore, erythroblasts with *H2AFY* isoform 1.1 knockdown showed a significant decrease in the CD71^+^CD235a^+^ intermediate erythroid cell population on day 11 (Figure 5D), followed by a decrease in the CD71^–^CD235a^+^ late erythroid cell population on day 14 compared with the scramble control (Figure 5E). Transduced progenitors with *H2AFY* isoform 1.1 knockdown also produced a significantly lower number of BFU-E colonies than did the scramble control (Figure 5F). Granulomonocytic cells with *H2AFY* isoform 1.1 knockdown (Figure 5, G and H) showed a significant increase in the CD14^+^CD15^+^ cell population compared with the scramble control (Figure 5I), and *H2AFY* isoform 1.1 knockdown exerted a skewing effect on granulomonocytic differentiation toward granulocytes (Figure 5J). Morphological examination confirmed the expansion of granulocyte eosinophils in granulomonocytic cultures with *H2AFY* isoform 1.1 knockdown compared with the scramble control on day 20 of culture (Figure 5K). Granulomonocytic cells with knockdown of the *H2AFY* isoform 1.1 showed increased apoptosis (Supplemental Figure 7C) and no significant cell-cycle changes (Supplemental Figure 7D). Our data indicate that *H2AFY* plays an important role in human hematopoiesis and that decreased expression of the isoform 1.1 expression associated with aberrant splicing of *H2AFY* in the presence of *U2AF1*^S34F^ impairs both erythroid and granulomonocytic differentiation.

**Figure 6:**
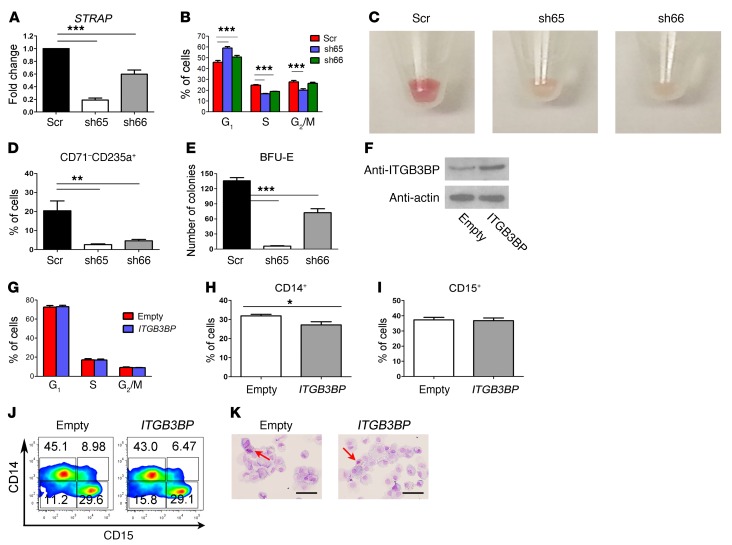
Knockdown of *STRAP* impairs erythroid differentiation, and overexpression of *ITGB3BP* is dispensable for granulomonocytic differentiation. (**A**) Expression levels of *STRAP* determined using qRT-PCR in erythroid cells with *STRAP* knockdown. (**B**) Cell-cycle analysis of erythroid cells on day 11 of culture. (**C**) Images of erythroid cell pellets on day 14 of culture for visual determination of hemoglobinization (*n* = 6). (**D**) Percentage of late erythroid (CD71^–^CD235a^+^) cells on day 14 of culture. (**E**) Number of BFU-E obtained from hematopoietic CD34^+^ progenitors with *STRAP* knockdown after 14 days in methylcellulose (colony-forming cell assays). (**F**) Western blots showing the expression levels of the ITGB3BP protein in granulomonocytic cells with *ITGB3BP* overexpression. (**G**) Cell-cycle analysis of granulomonocytic cells on day 11 of culture. (**H**) Percentage of CD14^+^ cells in granulomonocytic cultures on day 20 of culture. (**I**) Percentage of CD15^+^ cells in granulomonocytic cultures on day 20 of culture. (**J**) Representative flow cytometric contour plots (from 6 independent experiments) showing expression of CD14 and CD15 on day 20 of culture. (**K**) Representative images of May-Grünwald-Giemsa–stained granulomonocytic cells on day 20 of culture (*n* = 6). The red arrows indicate eosinophils. Scale bars: 25 μm. Results in each bar graph in panels **A**, **B**, **D**, **E**, and **G**–**I** were obtained from 6 independent experiments. Data represent the mean ± SEM. *P* values in panels **A**, **B**, **D**, and **E** were calculated by repeated-measures 1-way ANOVA with Tukey’s post-hoc test. *P* values in panels **H** and **I** were calculated by a paired, 2-tailed *t* test. *P < 0.05, **P < 0.01, and ***P < 0.001.

In this study, we showed that *U2AF1*^S34F^ induced skipping of exon 2 of the *STRAP* gene preferentially in erythroid colonies compared with granulomonocytic colonies (Figure 4, D and E). This splicing alteration gives rise to a premature stop codon, which is expected to lead to degradation of the mRNA transcripts by nonsense-mediated decay. Indeed, we found decreased expression of *STRAP* mRNA in *U2AF1*^S34F^ erythroid cells by qRT-PCR (Supplemental Figure 7E). In contrast, downregulation of *STRAP* mRNA was not observed in *U2AF1*^S34F^ granulomonocytic cells (Supplemental Figure 7F). To investigate the effects of this lineage-specific splicing alteration on hematopoiesis, shRNAs were used to knock down *STRAP* expression in bone marrow CD34^+^ progenitor cells differentiated toward the erythroid lineage (Figure 6A). One shRNA (sh66) resulted in approximately 50% knockdown (Figure 6A), a level similar to that observed in *U2AF1*^S34F^ erythroid cells (Supplemental Figure 7E). Erythroblasts with *STRAP* knockdown showed G_1_ cell-cycle arrest compared with the scramble control (Figure 6B). Similar to *U2AF1*^S34F^ erythroblasts, erythroblasts with *STRAP* knockdown showed defective hemoglobinization on day 14 (Figure 6C) and a significant decrease in the CD71^–^CD235a^+^ late erythroid cell population on day 14 of culture compared with the scramble control (Figure 6D). Transduced progenitors with *STRAP* knockdown also produced a significantly lower number of BFU-E colonies than did the scramble control (Figure 6E). These results support a critical role for *STRAP* in human erythroid differentiation.

Inclusion of exon 2 of *ITGB3BP* was associated with the presence of *U2AF1*^S34F^ in granulomonocytic cells only in our study. To investigate the effect of this splicing alteration on hematopoiesis, the isoform of *ITGB3BP* including exon 2 was overexpressed in bone marrow CD34^+^ progenitor cells differentiated toward the granulomonocytic lineage (Figure 6F). However, granulomonocytic cells with *ITGB3BP* overexpression showed no significant difference in cell-cycle pattern (Figure 6G) or apoptosis (Supplemental Figure 7G). We observed only a small reduction in the CD14^+^ monocytic cell population (Figure 6, H and J) and no difference in the CD15*^+^* granulocytic cell population (Figure 6, I and J) in cells with *ITGB3BP* overexpression compared with the EV control. Morphological examination confirmed that no skewing in granulomonocytic differentiation occurred in cells with *ITGB3BP* overexpression (Figure 6K). These results indicate that overexpression of the isoform of *ITGB3BP* including exon 2, while associated with the presence of *U2AF1*^S34F^ in granulomonocytic cells, does not significantly impact human granulomonocytic differentiation.

*Overexpression of U2AF1*^WT^*in U2AF1^S34F^–mutant MDS hematopoietic progenitors*. To investigate whether overexpression of U2AF1^WT^ rescues the aberrant hematopoiesis associated with U2AF1^S34F^ in MDS, we overexpressed U2AF1^WT^ in *U2AF1*^S34F^ MDS hematopoietic progenitors by retroviral transduction. Overexpression of U2AF1^WT^ in transduced *U2AF1*^S34F^ MDS erythroid and granulomonocytic cells was confirmed on day 11 by Western blotting (Supplemental Figure 8A). However, we observed no significant improvement in erythroid or granulomonocytic differentiation in transduced *U2AF1*^S34F^ MDS cells with U2AF1^WT^ overexpression compared with the EV control (Supplemental Figure 8, B–D). Importantly, we found that overexpression of U2AF1^WT^ did not correct the aberrant splicing activity of *H2AFY* or *STRAP* in *U2AF1*^S34F^ MDS cells (Supplemental Figure 8, E–G), consistent with the gain-of-function/neomorphic role of *U2AF1*^S34F^.

### Overexpression of H2AFY isoform 1.1 and the STRAP long isoform rescues erythroid differentiation defects in U2AF1^S34F^ MDS cells.

In order to determine whether modulation of *H2AFY* and *STRAP* isoform ratios can rescue the aberrant hematopoiesis associated with U2AF1^S34F^, we performed rescue experiments in which *H2AFY* isoform 1.1 or the *STRAP* long isoform were overexpressed in *U2AF1*^S34F^ MDS hematopoietic progenitors.

**Figure 7:**
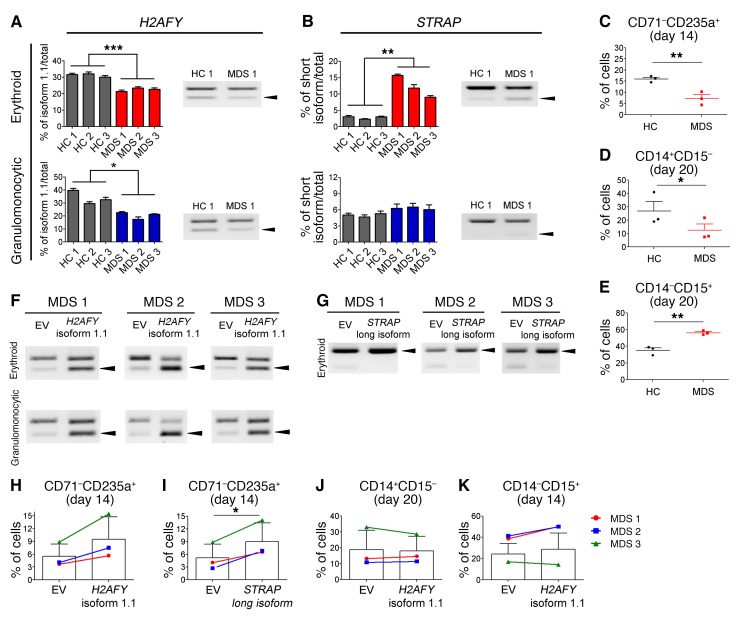
Effects of *H2AFY* isoform 1.1 and *STRAP* long isoform overexpression on erythroid and granulomonocytic differentiation in hematopoietic progenitors from *U2AF1*^S34F^ MDS patients. (**A**) *H2AFY* isoform 1.1 ratio in *U2AF1*^S34F^ MDS–differentiated erythroblasts and granulomonocytic cells (day 7 in culture) compared with that in healthy controls (HC). Arrowheads in blot indicate *H2AFY* isoform 1.1. (**B**) *STRAP* short isoform ratio in *U2AF1*^S34F^ MDS–differentiated erythroblasts (day 7 in culture) compared with healthy controls. Arrowheads in blot indicate the *STRAP* short isoform. (**C**–**E**) Impaired erythropoiesis and skewed differentiation toward granulocytes in *U2AF1*^S34F^ MDS hematopoietic progenitors compared with healthy controls. (**C**) Late erythroid (CD71^–^CD235a^+^) cell population on day 14 of culture and (**D**) monocytic (CD14^+^CD15^–^) and (**E**) granulocytic (CD14^–^CD15^+^) cell populations on day 20 of culture were measured by flow cytometry. (**F** and **G**) Overexpression of (**F**) *H2AFY* isoform 1.1 and (**G**) *STRAP* long isoform in *U2AF1*^S34F^ MDS hematopoietic progenitors differentiating toward erythroid and granulomonocytic lineages. Arrowheads in blots indicate *H2AFY* isoform 1.1 or the *STRAP* short isoform. (**H**–**K**) Effects of *H2AFY* isoform 1.1 and *STRAP* long isoform overexpression on erythroid and granulomonocytic differentiation of *U2AF1*^S34F^ MDS hematopoietic progenitors. Late erythroid (CD71^–^CD235a^+^) cell population in transduced erythroblasts expressing *H2AFY* isoform 1.1 (**H**) or the *STRAP* long isoform (**I**), measured by flow cytometry on day 14 of culture compared with the EV control. (**J**) Monocytic (CD14^+^CD15^–^) and (**K**) granulocytic (CD14^–^CD15^+^) cell populations in transduced granulomonocytic cells expressing *H2AFY* isoform 1.1 were measured by flow cytometry on day 20 of culture compared with the EV control. In panels **A** and **B**, quantification of altered splicing events in gel was performed using ImageJ. Bar graph results in **A** and **B** were obtained from 4 technical replicates. Data represent the mean ± SEM. *P* values in panels **A**–**E** were calculated by an unpaired, 2-tailed *t* test. *P* values in panels **H**–**K** were calculated by a paired, 2-tailed *t* test. *P < 0.05, **P < 0.01, and ***P < 0.001.

First, we evaluated whether aberrant splicing of *H2AFY* and *STRAP* occurs in the erythroid and granulomonocytic cells differentiated from *U2AF1*^S34F^ MDS hematopoietic progenitors. We differentiated *U2AF1*^S34F^ MDS CD34^+^ hematopoietic progenitor cells into erythroid and granulomonocytic cells as previously described (27, 28). Consistent with our RNA-seq results of *U2AF1*^S34F^-transduced erythroid and granulomonocytic colonies (Figure 4), *U2AF1*^S34F^ MDS erythroid and granulomonocytic cells harvested on day 7 showed a significant reduction in *H2AFY* isoform 1.1 compared with healthy controls (Figure 7A). *U2AF1*^S34F^ MDS erythroid cells harvested on day 7 also demonstrated a significant increase in the *STRAP* short isoform compared with healthy controls (Figure 7B). We did not observe aberrant splicing of *STRAP* in *U2AF1*^S34F^ MDS granulomonocytic cells harvested on day 7, further confirming our results of a lineage-specific effect of *U2AF1*^S34F^ mutation on this target gene (Figure 7B). Moreover, *U2AF1*^S34F^ MDS hematopoietic progenitors showed impaired erythroid differentiation on day 14 (Figure 7C) and skewed granulomonocytic differentiation toward granulocytes on day 20 (Figure 7, D and E) compared with healthy control cells.

Next, we overexpressed *H2AFY* isoform 1.1 or the *STRAP* long isoform in *U2AF1*^S34F^ MDS hematopoietic progenitors by lentiviral transduction. Overexpression of *H2AFY* isoform 1.1 in transduced *U2AF1*^S34F^ MDS erythroid and granulomonocytic cells was confirmed by RT-PCR compared with the EV control (Figure 7F). We also confirmed overexpression of the *STRAP* long isoform in transduced *U2AF1*^S34F^ MDS erythroblasts by RT-PCR compared with the EV control (Figure 7G). The percentage of CD71^–^CD235a^+^ erythroblasts on day 14 ranged from 14.5% to 17.2% in healthy controls (Figure 7C). Overexpression of *H2AFY* isoform 1.1 resulted in an increase in late CD71^–^CD235a^+^ erythroblasts (to 5.7% to 15.5%, i.e., a 1.54- to 1.85-fold increase) on day 14 in the three *U2AF1*^S34F^ MDS patients, with a trend toward significance compared with the EV control (Figure 7H). Overexpression of the *STRAP* long isoform resulted in a significant increase in late CD71^–^CD235a^+^ erythroblasts (to 6.5% to 14.1%, i.e., a 1.59- to 2.53-fold increase) on day 14 in the three *U2AF1*^S34F^ MDS patients compared with the EV control (Figure 7I). However, we observed no change in monocytic or granulocytic cell differentiation in transduced *U2AF1*^S34F^ MDS granulomonocytic cells with overexpression of *H2AFY* isoform 1.1 (Figure 7, J and K).

These results indicate that overexpression of *H2AFY* isoform 1.1 and the *STRAP* long isoform rescues erythroid differentiation defects in *U2AF1*^S34F^ MDS cells.

## Discussion

Patients with MDS suffer from refractory anemia, which is a defining feature of this disorder (1, 33). In our study, we demonstrated that expression of *U2AF1*^S34F^ in human hematopoietic progenitors results in impaired erythroid differentiation as a result of poor hemoglobinization and reduced growth of erythroid progenitors. These in vitro results therefore show that the presence of the *U2AF1* mutation impairs human erythropoiesis and indicate that this mutation may play an important role in the development of anemia in MDS. Interestingly, it has been reported that *U2AF1* mutations in patients with MDS are associated with lower hemoglobin levels when compared with patients with WT *U2AF1* (34).

MDS have a defective maturation program of myeloid progenitors (1, 4). We showed that expression of *U2AF1*^S34F^ in human hematopoietic progenitors results in the skewing of granulomonocytic differentiation toward granulocytes (specifically eosinophils). Similar phenotypes were also observed in the bone marrow compartment of an *U2AF1*^S34F^-transgenic mouse model (26): *U2AF1*^S34F^ mice showed a significant decrease in monocytes, together with a significant increase in granulocyte neutrophils in the bone marrow, compared with control mice (26). Interestingly, eosinophilia has been reported in some patients with de novo MDS (35, 36). Our study demonstrates a link between *U2AF1*^S34F^ and skewed granulomonocytic differentiation in human hematopoiesis.

We found that expression of *U2AF1*^S34F^ has a suppressive effect on cell growth in transduced erythroid and granulomonocytic cells. Likewise, introduction of the *U2AF1*^S34F^ mutation into cancer cell lines results in impaired cell growth (6), and this is also observed with other splicing factor gene mutations (37). How splicing factor mutations confer a clonal growth advantage in myeloid malignancies is not yet fully understood.

The phenotypic changes produced by *U2AF1*^S34F^ likely reflect differences in the downstream target genes and pathways affected in each lineage and in the type of aberrant splicing events associated with the presence of *U2AF1*^S34F^. In support of this, we identified aberrant splicing events in many downstream target genes specific to *U2AF1*^S34F^ erythroid and granulomonocytic colonies, using RNA-seq. Our RNA-seq data should be interpreted with the caveats that *U2AF1*^S34F^ expression levels (at a variant allele frequency [VAF] of >80%) in *U2AF1*^S34F^-transduced cells is higher than the level expected in MDS patients with heterozygous *U2AF1* mutations (i.e., 50% VAF) and that the *U2AF1*^S34F^ erythroid and granulomonocytic colonies analyzed contain some heterogeneity in the stage of differentiation (but they are nevertheless all fully committed toward either the myeloid or erythroid path). We suggest that the aberrantly spliced target genes may play a role in the aberrant growth and differentiation of cells of the erythroid and granulomonocytic lineages expressing this mutation.

Other studies have shown that aberrant cassette exon splicing is a common consequence of *U2AF1* mutations (18, 23, 25), and we found that cassette exons were the most common type of aberrant splicing event induced by *U2AF1*^S34F^ in both erythroid and granulomonocytic colonies. Interestingly, we observed a significantly higher number of misregulated cassette exon events in granulomonocytic *U2AF1*^S34F^ colonies compared with erythroid *U2AF1*^S34F^ colonies in our study (Figure 3, B and C). Notably, the UAG 3′ splice site signature of cassette exons that are more skipped is similar to the optimal RNA-binding sequence for the U2AF heterodimer (16), strongly supporting the idea that missplicing of this group of exons is a direct consequence of *U2AF1* mutation. The fact that these exons have significantly weaker 5′ splice sites suggests that their inclusion may be dependent on optimal interaction of U2AF1 at their 3′ splice sites, explaining their sensitivity to *U2AF1* mutation. Splicing of many exons is dependent not only on the strength of their consensus splice site sequences, but also on auxiliary elements such as exonic splicing enhancers (ESEs) (38). There are numerous classes of ESEs that bind different splicing activators often expressed with different cell-type specificity. Lineage- and exon-specific skipping might therefore occur in the absence of the optimal U2AF1–3′ splice site interaction (*U2AF1*^S34F^), combined with a cell type–specific lack of the ESE-binding activator protein. In contrast, the molecular basis of the increased inclusion of other cassette exons in response to *U2AF1*^S34F^ is unclear, although it was observed previously that some exons are upregulated upon knockdown of U2AF1 (39).

In our study, we found that 20 genes were aberrantly spliced in cells of both erythroid and granulomonocytic lineages and thus represent common downstream targets of *U2AF1*^S34F^ that may play a role in the phenotypic changes observed in both lineages. *H2AFY* was one of these genes, showing a mutually exclusive exon splicing event in both lineages. *H2AFY* encodes the core histone macro-H2A1, which is involved in X chromosome inactivation (40) and is required for both transcriptional silencing and induction (41). Intriguingly, loss of X chromosome inactivation results in MDS-like phenotypes in mice (42). Alternative splicing in *H2AFY* has been shown to generate 2 functionally different isoforms (43). A role for macro histone variants in repressing gene expression during cell differentiation has been shown in embryonic and adult stem cells, and in pluripotent cells, in which these variants are recruited to the regulatory regions of genes that retain or gain H3K27me3 during differentiation (44). *H2AFY* isoform 1.1 has been shown to be generally expressed in more differentiated cells (45). We found a reduction in the expression of isoform 1.1 of *H2AFY* associated with *U2AF1*^S34F^ in transduced erythroid cells. Critically, we then showed that knockdown of *H2AFY* isoform 1.1 in human erythroblasts resulted in impaired erythroid differentiation. Interestingly, isoform changes in *H2AFY* have been shown to play a role in normal erythroid differentiation. Pimentel et al. demonstrated that the relative expression of mutually exclusive exons is reversed almost completely between proerythroblasts (mostly expressing isoform 1.2) and orthochromatic erythroblasts (mostly expressing isoform 1.1), indicating that the alternative splicing switch from isoform 1.2 to isoform 1.1 of *H2AFY* occurs during normal late erythroid differentiation (46). Our data show that aberrant splicing of *H2AFY* associated with the presence of *U2AF1*^S34F^ leads to a reduction in the expression of isoform 1.1 of this gene. Here, isoform 1.1 is the minor isoform (compared with isoform 1.2), and, strikingly a small reduction in this isoform resulted in a marked impairment of erythropoiesis. We provide evidence that this imbalance of the two *H2AFY* isoforms prevents erythroblasts from undergoing terminal differentiation. We suggest that aberrant splicing of *H2AFY* leads to impaired erythropoiesis and may play a role in the anemia observed in MDS patients with *U2AF1* mutation.

We also found a reduction in the expression of isoform 1.1 of *H2AFY* associated with *U2AF1*^S34F^ in granulomonocytic cells. Knockdown of the *H2AFY* isoform 1.1 in granulomonocytic cells resulted in a skewed differentiation toward the granulocyte population, specifically eosinophils, closely mirroring the effects of *U2AF1*^S34F^ on granulomonocytic differentiation. Our functional data therefore indicate that changes in the abundance of *H2AFY* isoform 1.1 also play a role in the differentiation of granulomonocytic cells. Thus, reduced expression of *H2AFY* isoform 1.1 recapitulated the phenotypes associated with expression of *U2AF1*^S34F^ in erythroid and/or granulomonocytic cells, suggesting a causal link. Importantly, we showed that the aberrant splicing event in the *H2AFY* gene that we identified in *U2AF1*^S34F^ erythroid and granulomonocytic colonies also occurs in purified bone marrow CD34^+^ cells from patients with *U2AF1*^S34F^ MDS.

We have shown that *U2AF1*^S34F^ differentially alters mRNA splicing of many target genes in a lineage-specific manner in erythroid and granulomonocytic colonies. For example, splicing alterations in *STRAP* and *SMARCA5* preferentially occur in the erythroid lineage, while splicing alterations in *ITGB3BP* and *ATR* preferentially occur in the granulomonocytic lineage. STRAP is a serine/threonine kinase receptor–associated protein that plays a role in the cellular distribution of the survival of motor neurons (SMN) complex, important in the assembly of snRNPs (47). Deregulated expression of *STRAP* is associated with other human cancers, including those of the lung, colon, and breast (48, 49).

In this study, we showed that *U2AF1*^S34F^ induced aberrant splicing of *STRAP*, leading to downregulation of this gene in erythroid cells only, and that knockdown of *STRAP* in human erythroblasts results in impaired erythroid differentiation. These results support a critical role for *STRAP* in human erythroid differentiation and suggest that aberrant splicing of *STRAP* leads to impaired erythropoiesis in association with *U2AF1*^S34F^. Interestingly, STRAP acts as a negative regulator of the TGF-β signaling pathway through interaction with Smad7 (50), and TGF-β signaling exerts an inhibitory effect on erythroid cell growth during differentiation (51).

We have thus identified *H2AFY* and *STRAP* as key downstream target genes of *U2AF1*^S34F^ in human cells of the myeloid lineage, with aberrantly spliced *H2AFY* and *STRAP* both being critical effectors of impaired erythropoiesis and aberrantly spliced *H2AFY* also being a critical effector of aberrant granulomonocytic cell differentiation. While aberrant splicing of these target genes leads to impaired differentiation of progenitor cells, in the stem cell compartment this might be predicted to result in a negative selection pressure on stem cells. We suggest that these splicing abnormalities play a role in the development of cytopenias that are hallmarks of MDS.

Importantly, we provide the first evidence, to our knowledge, that isoform modulation of the *U2AF1*^S34F^ target genes *H2AFY* and *STRAP* ameliorates the erythroid differentiation defect in *U2AF1*^S34F^ MDS cells. Overexpression of *H2AFY* isoform 1.1 and the *STRAP* long isoform resulted in an improvement in erythroid differentiation, raising the possibility of using splicing modulators therapeutically. Approaches that involve the use of antisense/splice site–switching oligonucleotides (ASOs/SSOs) alter the balance between mRNA isoforms, with the aim of restoring normal splicing or preferentially expressing specific isoforms (52). ASOs/SSOs have shown efficacy in splicing modulation in in vivo mouse studies and are being used in clinical trials for the treatment of spinal muscular atrophy and Duchenne muscular dystrophy (53). The use of splicing modulators may have efficacy in the treatment of myeloid malignancies with *U2AF1* mutations.

We observed no change in granulomonocytic differentiation in transduced *U2AF1*^S34F^ MDS cells overexpressing *H2AFY* isoform 1.1, indicating that the correction of a single isoform change of *H2AFY* may not be sufficient to fully rescue the defect in granulomonocytic differentiation in patients’ cells and that modulation of aberrant splicing of multiple target genes may be required.

We also overexpressed *U2AF1*^WT^ in *U2AF1*^S34F^ MDS hematopoietic progenitors. However, we observed no significant difference in erythroid or granulomonocytic differentiation between transduced *U2AF1*^S34F^ MDS cells with *U2AF1*^WT^ overexpression and EV controls. *U2AF1* mutations in myeloid malignancies are considered gain-of-function/neomorphic mutations. As *U2AF1*^S34F^ is still expressed in MDS cells, it would continue to cause aberrant splicing of its target genes, and this may therefore explain why overexpression of *U2AF1*^WT^ does not rescue the functional defects observed in *U2AF1*^S34F^ MDS cells. Alternatively, the U2AF1 mutation may exert its effect mostly early in MDS disease development, and the rescue is less effective at a later stage.

Our results demonstrate that *U2AF1*^S34F^ shows lineage specificity in altering pre-mRNA splicing of downstream target genes, resulting in different phenotypes in different myeloid lineages. These findings shed light on the events underlying the phenotypic heterogeneity in MDS. It will be important to determine whether other splicing factor genes commonly mutated in MDS, namely *SF3B1*, *SRSF2*, and *ZRSR2*, also exhibit lineage specificity in altering the splicing of target genes to drive different phenotypes in different hematopoietic lineages.

The identification of key target genes of the common spliceosome mutations in cells of the erythroid and granulomonocytic lineages is crucial for understanding how the mutations contribute to MDS pathophysiology and for designing new targeted therapeutic strategies. It is now recognized that splicing factor gene mutations are common in many cancers, and we believe that our work has broad implications for the understanding of how these mutations result in cell type–specific phenotypes in other malignancies.

## Methods

### Cell culture.

Bone marrow CD34^+^ cells from healthy controls (Lonza) and from *U2AF1*^S34F^ MDS patients were cultured for 14 and 20 days to generate erythroblasts and granulomonocytic cells, respectively, as previously described (27, 28). All cytokines were obtained from Miltenyi Biotec, except for erythropoietin, which was obtained from Roche. Medium was replenished every second day to maintain the same cell concentration.

### Viral transduction.

Retroviral pGCDNsam-IRES-EGFP plasmids containing *U2AF1*^S34F^ and *U2AF1*^WT^ cDNA were used following replacement of the EGFP sequence with neomycin to impart G418 resistance (6). To obtain high-titer retrovirus stock, vector plasmids were cotransfected with vesicular stomatitis virus glycoprotein envelope and Gag/Pol plasmids using Lipofectamine 2000 (Life Technologies, Thermo Fisher Scientific) into HEK293T cells as previously described (6). Spinoculation was performed in the presence of 8 μg/ml polybrene (Sigma-Aldrich) at 800 ×*g* and 32°C for 2 hours. After overnight incubation with retroviruses, the selection of transduced cells was performed in medium containing Geneticin (Life Technologies, Thermo Fisher Scientific) (0.75 mg/ml and 1.0 mg/ml for erythroid and granulomonocytic cells, respectively).

Lentiviral constructs containing shRNAs targeting *H2AFY* isoform 1.1 were obtained by cloning oligonucleotides (shRNA sequences are described in Supplemental Table 1) into the pLKO.1-puro vector (Sigma-Aldrich). shRNAs targeting *STRAP* in the pLKO.1-puro vector (TRCN0000060465 and TRCN0000060466) were obtained from Sigma Mission. The lentiviral construct containing *ITGB3BP* isoform 1, *H2AFY* isoform 1.1, or *STRAP* long isoform cDNA in the pReceiver-Lv156 vector was obtained from GeneCopoeia. The procedures of lentivirus production and transduction were the same as those used for retroviruses, except MISSION lentiviral packaging mix (Sigma-Aldrich) was used for transfection with vector plasmids into HEK293T cells. The selection of transduced cells was performed in medium containing 0.65 μg/ml puromycin (Thermo Fisher Scientific).

### Flow cytometry.

To evaluate erythroid differentiation, cells were incubated for 30 minutes on ice with anti-CD36-PE (clone 5-271; BioLegend), anti-CD71-FITC (clone OKT9; eBioscience), and anti–CD235a-APC (clone HIR2; BioLegend) antibodies before staining with 0.5 μg/ml DAPI (Sigma-Aldrich). To evaluate granulomonocytic differentiation, cells were first incubated for 30 minutes on ice with Fixable Viability Dye eFluor 780 (eBioscience). After washing with PBS, cells were incubated on ice for 30 minutes with anti-CD11b Brilliant Violet 421 (clone ICRF44; BioLegend), anti–CD14-FITC (clone RMO52; Beckman Coulter), and anti–CD15-APC (clone W6D3; BioLegend) antibodies. To perform apoptosis assays, cells resuspended in annexin V–binding buffer (BioLegend) were stained with annexin V-FITC antibody (BioLegend) for 20 minutes in the dark according to the manufacturer’s instructions. Propidium iodide (1 μg/ml) was added to the cells before analysis. To perform cell-cycle analysis, cells were fixed with ice-cold absolute ethanol for 30 minutes before incubation with 40 μg/ml propidium iodide and 10 μg/ml RNase A for 1 hour at 37°C. Flow cytometry was performed on a BD LSRII (BD Biosciences), and the data were analyzed using FlowJo software, version 7.6.4.

### Colony-forming cell assays.

Colony-forming cell assays were performed using MethoCult H4434 and H4534 methylcellulose (STEMCELL Technologies) according to the manufacturer’s instructions. Transduced cells (3,000 cells) were grown in methylcellulose containing 1.0 mg/ml Geneticin (Thermo Fisher Scientific). The number and morphology of the colonies were analyzed after 14 days in culture.

### RNA-seq.

Individual BFU-E colonies (*n* = 3 each for *U2AF1*^S34F^, *U2AF1*^WT^, and EV) and CFU-G/M colonies (*n* = 3 each for *U2AF1*^S34F^, *U2AF1*^WT^, and EV) were first harvested for RNA extraction using TRIzol reagent (Thermo Fisher Scientific) according to the manufacturer’s instructions. RNA was also extracted from bone marrow CD34^+^ cells enriched from mononuclear cells obtained from 6 MDS patients and 5 healthy controls using CD34 MicroBeads (Miltenyi Biotec). Two patients with MDS had *U2AF1*^S34F^ mutations, whereas 4 patients had no known mutations in splicing factor genes (*SF3B1, SRSF2, U2AF1*, or *ZRSR2)* as determined by targeted next-generation sequencing data from a previous study (5). Linear acrylamide (20 μg) (Thermo Fisher Scientific) was used as the RNA coprecipitant. Total RNA was then DNase treated (Invitrogen, Thermo Fisher Scientific), purified using XP beads (Beckman Coulter), and amplified (100  ng) for 12 cycles using the SMART mRNA Amplification Kit (Clontech) according to the manufacturer’s instructions. Library preparation was performed using the NEBNext DNA Library Prep Kit (New England BioLabs) according to the manufacturer’s instruction. Illumina universal paired-end adapters were used. Custom indexes were designed in house to barcode sequences for multiplexing. Sequencing was performed using the Illumina HiSeq 2500 platform (100-bp read length, paired-end) and the Reagent Kit, version 3 (Illumina).

### Mapping, filtering, and alternative splicing analysis.

Following quality control (QC) analysis with the fastQC package (http://www.bioinformatics.babraham.ac.uk/projects/fastqc), reads were aligned using STAR (54) against the human genome assembly (NCBI build37 [hg19] UCSC transcripts). QC was performed on the mapped files using RNA-SeQC (55) (Supplemental Table 2). Nonuniquely mapped reads and reads that were identified as PCR duplicates using Samtools (56) were discarded. Gene expression levels were quantified as read counts using the featureCounts function (57) from the Subread package (58) with default parameters, and the RPKM values were generated using the edgeR package (59). The aligned reads were reconstructed into transcripts using Cufflinks (cuffmerge) (60) to produce a reference-guided assembly (with NCBI build37 [hg19] UCSC transcripts). Alternative 3′ and 5′ splice sites, skipped (cassette) exons, mutually exclusive exons, and retained introns were quantified using rMATS (31), with the assembly produced from Cufflinks. Splicing events were considered significantly different in *U2AF1*^S34F^ compared with the *U2AF1*^WT^ or EV control if they met the criteria of a FDR of 0.05 or less, a change in inclusion level of 10% or higher (23), and did not appear as significant events in the comparison between *U2AF1*^WT^ and EV (see Supplemental data file 1). The alternative spliced events were then plotted using the sashimi plots from the Mixture of Isoforms (MISO) software (61). The results were visualized and filtered using the data visualization tool Zegami (http://zegami.com/). Integrative Genomics Viewer (IGV) version 2.3 (http://www.broadinstitute.org/igv/) was used for visualization of the sequence reads. The data discussed in this article have been deposited in the NCBI’s Gene Expression Omnibus (GEO) database (GEO GSE94153).

### Gene ontology analysis.

Gene ontology analysis of the RNA-seq data was performed using GOseq (62). A weighted bias correction based on the number of exons in each gene from the NCBI build37 (hg19) UCSC transcripts was applied.

### Isoform-specific qRT-PCR and RT-PCR.

To determine the expression levels of specific isoforms of *H2AFY*, *STRAP*, *SMARCA5*, *ITGB3BP*, and *ATR*, primers were designed to span the region affected by aberrant splicing induced by *U2AF1*^S34F^ (Supplemental Table 1). Expression levels of these isoforms were measured by qRT-PCR using LightCycler 480 SYBR Green I Master Mix (Roche) according to the manufacturer’s instructions. The *GAPDH* gene was used to normalize for differences in input cDNA using PrimePCR SYBR Green Assay: *GAPDH*, Human (Bio-Rad). Each sample was run in triplicate, and the expression ratios were calculated using the ΔΔCt method. To confirm the splicing events of *H2AFY* and *STRAP*, RT-PCR was performed to amplify the region affected by aberrant splicing. Quantification of the different isoforms was achieved by agarose gel electrophoresis and ImageJ (NIH) analysis.

### Analysis of cassette exon properties.

For analysis of cassette exon properties, we used “skipped exons” (SE) from the rMATS output with a FDR of less than 0.05, and an “IncLevelDifference” of less than –0.10 (“more skipped”) or greater than 0.10 (“more included”). For each SE, we extracted sequences corresponding to its 3′splice site and 5′splice site as well as sequences corresponding to the 5′splice site of the upstream exon and 3′ splice site of the downstream exon. For the erythroid *U2AF1*^S34F^ versus *U2AF1*^WT^ cells, we obtained 71 exons that were more skipped and 108 exons that were more included. For the granulomonocytic *U2AF1*^S34F^ versus *U2AF1*^WT^ cells, we obtained 190 more skipped exons and 157 more included exons. As control data sets, we used SE exons expressed in the erythroid and granulomonocytic cells, but not regulated (FDR >0.05 and IncLevelDifference values of –0.05 to 0.05). Human sequences were retrieved from the UCSC Genome Browser (hg19, February 2009) using R and the following Bioconductor packages: Genomic Ranges, Genomic Features, biomaRt, and BSgenome.Hsapiens.UCSC.hg19 (63–65). Graphical outputs were generated with the CRAN package ggplot2. For box plots, the whiskers represent 1.5 times the interquartile range. Statistical analysis comparing sequences properties between data sets was done using a Kruskal-Wallis test, followed by 2-sided Mann-Whitney *U* tests with Bonferroni’s correction. Sequence logos were produced using WebLogo (http://weblogo.berkeley.edu/logo.cgi) (66). Splice site strength was calculated using MaxEntScan software (http://genes.mit.edu/burgelab/maxent/Xmaxentscan_scoreseq_acc.html) (67). Sequences were extracted following the developer’s instructions for 3′ splice sites and 5′splice sites. MaxEnt scores were plotted using ggplot2 (http://github.com/hadley/ggplot2). Branch point scores and the distance the between branch point and the 3′splice site was obtained using SVM-BPfinder (http://regulatorygenomics.upf.edu/Software/SVM_BP/) (68). Data were filtered by restricting the predicted branch point to being located between the 3′splice site and 12 bp upstream of the AG-dinucleotide exclusion zone (AGEZ) and selecting the top svm_scr scoring for each event.

### Statistics.

The significance of the comparisons between cells transduced with *U2AF1*^S34F^, *U2AF1*^WT^, or EV was determined by repeated-measures 1-way ANOVA with Tukey’s post-hoc tests. The measurements of cell growth over time for cells transduced with *U2AF1*^S34F^, *U2AF1*^WT^, or EV were compared using 2-way ANOVA and Bonferroni’s post tests. The significance of comparisons between 2 groups was determined by paired or unpaired 2-tailed *t* tests as indicated. Comparisons of each type of aberrantly spliced events identified by rMATS between erythroid and granulomyocytic conditions were determined by Fisher’s exact test with Bonferroni’s correction. *P* values of less than 0.05 were considered statistically significant.

### Study approval.

The MDS patient samples used in this study were obtained with written informed consent under approval by the IRBs of the Karolinska Institutet and the IRCSS Policlinico San Matteo.

Information regarding qRT-PCR, Western blotting, cell growth assays, May-Grünwald–Giemsa staining, pyrosequencing, SYBR Green qRT-PCR, and cloning and Sanger sequencing can be found in the Supplemental Methods.

## Author contributions

BHY, VS, RNA, SR, JS, HD, and MB conducted experiments. BHY, ER, ML, ST, AV, PV, NCPC, CWJS, AP, and JB analyzed data. LM, MC, EHL, and SO provided essential samples or reagents for the study. BHY, CWJS, AP, and JB designed the study. BHY, ER, ML, CWJS, AP, and JB wrote the manuscript.

## Supplementary Material

Supplemental data

Supplemental data file 1

Supplemental data file 2

Supplemental data file 3

Supplemental data file 4

Supplemental data file 5

Supplemental data file 6

Supplemental data file 7

Supplemental data file 8
