# Supplementary material for: The U2AF1S34F mutation induces lineage-specific splicing alterations in myelodysplastic syndromes
Source: J Clin Invest. 2017 Apr 24;127(6):2206–21. doi: 10.1172/JCI91363 (PMC5451246; doi:10.1172/JCI91363)
Supplement: Supplemental data [file jci-127-91363-s001.pdf]

The *U2AF1*^{S34F} mutation induces lineage-specific splicing alterations in myelodysplastic syndromes

Bon Ham Yip, Violetta Steeples, Emmanouela Repapi, Richard N. Armstrong, Miriam Llorian, Swagata Roy, Jacqueline Shaw, Hamid Dolatshad, Stephen Taylor, Amit Verma, Matthias Bartenstein, Paresh Vyas, Nicholas C. P. Cross, Luca Malcovati, Mario Cazzola, Eva Hellström-Lindberg, Seishi Ogawa, Christopher W. J. Smith, Andrea Pellagatti, Jacqueline Boultwood

Supplementary Information

Supplemental Methods

Real-time quantitative PCR

The expression level of *U2AF1* was determined by real-time quantitative PCR. The *β2-microglobulin* gene was used to normalize for differences in input cDNA. Pre-developed TaqMan Assays were used (Assays-on-Demand, Applied Biosystems, Foster City, CA, USA) and reactions were run on a LightCycler 96 Real-Time PCR System (Roche). Each sample was run in triplicate and the expression ratios were calculated using the $\Delta\Delta C_T$ method.

Western blot

Western blot was performed using the Invitrogen NuPage Novex 4–12% Bis-Tris Gels as previously described (1). Anti-FLAG M2-Peroxidase (HRP) antibody (Sigma Aldrich), anti-U2AF35 antibody (Abcam ab86305), anti-ITGB3BP antibody (HPA028463; Atlas antibodies), and anti-beta actin antibody (HRP) (Abcam ab197277) at 1:2500, 1:2000, 1:500 and 1:30000 dilution respectively were used.

Cell Growth Assay

Transduced cells on day 8 were seeded into 96-well plates (20,000 cells/0.2 mL) and viable cell counts were determined by trypan blue exclusion for 6 consecutive days. Medium was replenished every second day to maintain the same volume.

May-Grünwald-Giemsa staining

Cytospin slides of cultured granulomonocytic cells were prepared and stained with May-Grünwald and Giemsa solution according to the manufacturer’s instructions (Sigma Aldrich).

Pyrosequencing

PCR and Sequencing primers were designed using PyroMark Assay Design 2.0 software and are shown in Supplemental Table 1. PCR of colony cDNA was performed with the PyroMark PCR kit (Qiagen) using the standard component mix (1.5mM $MgCl_2$) and thermocycling conditions (55°C annealing temperature). Pyrosequencing was performed on a PyroMark Q24 instrument (Qiagen) according to the manufacturer's recommendations.

SYBR green real-time qPCR

Primers described in Park et al. (2) were used to perform a SYBR green real-time qPCR to assess ATG7 polyadenylation site usage. Samples were run on a Roche Lightcycler 96 using Roche lightcycler 480 SYBR green I master according to the manufacturer's protocol.

Cloning and Sanger sequencing

The coding sequence of the *H2AFY* and *STRAP* genes were amplified from cDNA obtained from erythroid and granulomonocytic colonies by PCR using Phusion high fidelity DNA polymerase (NEB). PCR products were purified using a QIA quick gel extraction kit (Qiagen) and A tailed using Maxima hot start PCR mastermix. PCR products were inserted into the pCR4-TOPO vector using a TOPO TA Cloning Kit (Life technologies) and transformed in DH5α chemically competent cells (Life technologies). These were grown at 37 °C on LB Agar plates supplemented with 100 µg/ml ampicillin (Sigma). Individual colonies were picked and expanded in LB medium with 100 µg/ml ampicillin, and plasmid DNA was then extracted using a Qiaprep spin miniprep kit (Qiagen). Plasmid insert sequences were obtained by Sanger sequencing (Source Bioscience) using M13F and M13R primers.

References

1. Yip BH, et al. Effects of L-leucine in 5q- syndrome and other RPS14-deficient erythroblasts. *Leukemia*. 2012;26(9):2154-2158.

2. Park SM, et al. U2AF35(S34F) Promotes Transformation by Directing Aberrant ATG7 Pre-mRNA 3' End Formation. *Mol Cell*. 2016;62(4):479-490.

Supplemental Figure 1

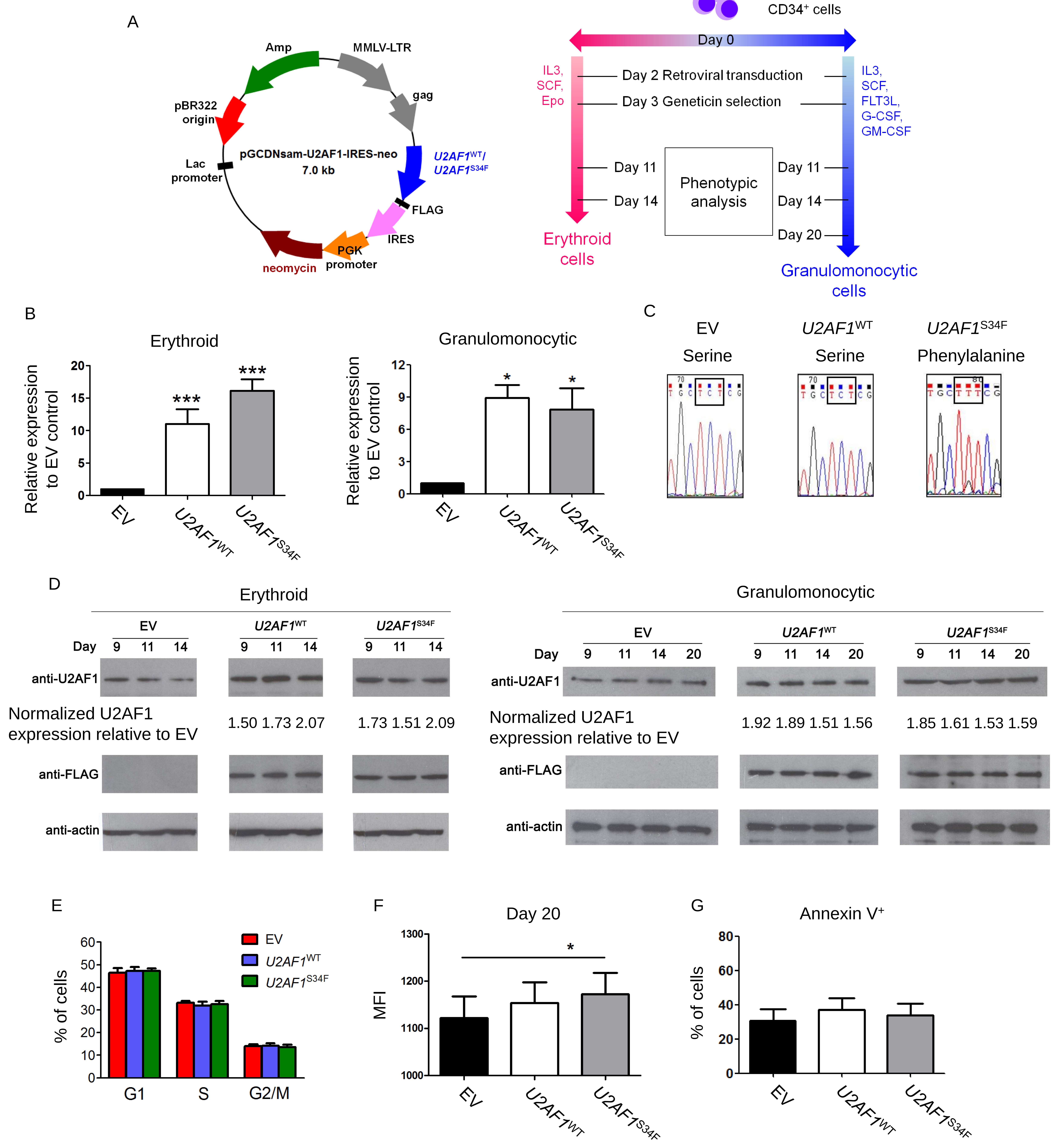

Supplemental Figure 1. Expression of *U2AF1*^{WT} and *U2AF1*^{S34F} in hematopoietic CD34^{+} progenitors. (A) Schematic diagram showing the retroviral pGCDNsam-IRES-neomycin plasmids containing *U2AF1*^{WT} or *U2AF1*^{S34F} cDNA (left). Schematic diagram showing the culture conditions used to obtain erythroid and granulomonocytic cells following retroviral transduction of a plasmid expressing *U2AF1*^{WT} or *U2AF1*^{S34F} cDNA into hematopoietic progenitors (right). (B) Taqman qRT-PCR to determine the relative expression levels of *U2AF1*^{WT} or *U2AF1*^{S34F} transcripts in transduced cells differentiating towards erythroid and granulomonocytic cells harvested on Day 11. Results in each bar graph were obtained from 6 independent experiments. (C) Sanger sequencing of cDNA from transduced cells confirming the expression of the *U2AF1*^{S34F} mutation. (D) Expression of U2AF1^{WT} and U2AF1^{S34F} at different time points in transduced erythroid and granulomonocytic cells in culture. Quantification of protein expression levels was performed by ImageJ. (E) Cell cycle analysis of transduced erythroid cells expressing U2AF1^{WT} or U2AF1^{S34F} on day 11 of culture. Results were obtained from 6 independent experiments. (F) Granulomonocytic differentiation in transduced granulomonocytic cells expressing U2AF1^{WT} or U2AF1^{S34F}. Median fluorescence intensity (MFI) of forward scatter (as a measure of cell size) of transduced granulomonocytic cells expressing U2AF1^{WT} or U2AF1^{S34F} on day 20 of culture. Results were obtained from 5 independent experiments. (G) Apoptosis in transduced granulomonocytic cells expressing U2AF1^{WT} or U2AF1^{S34F}. Apoptosis was measured by Annexin V staining and flow cytometry in transduced granulomonocytic cells expressing U2AF1^{WT} or U2AF1^{S34F} on day 11 of culture. Results were obtained from 7 independent experiments. Bar graphs show mean+SEM. *$P<0.05$, **$P<0.01$ and ***$P<0.001$, 1-way ANOVA with repeated measures using Tukey's post-test.

Supplemental Figure 2

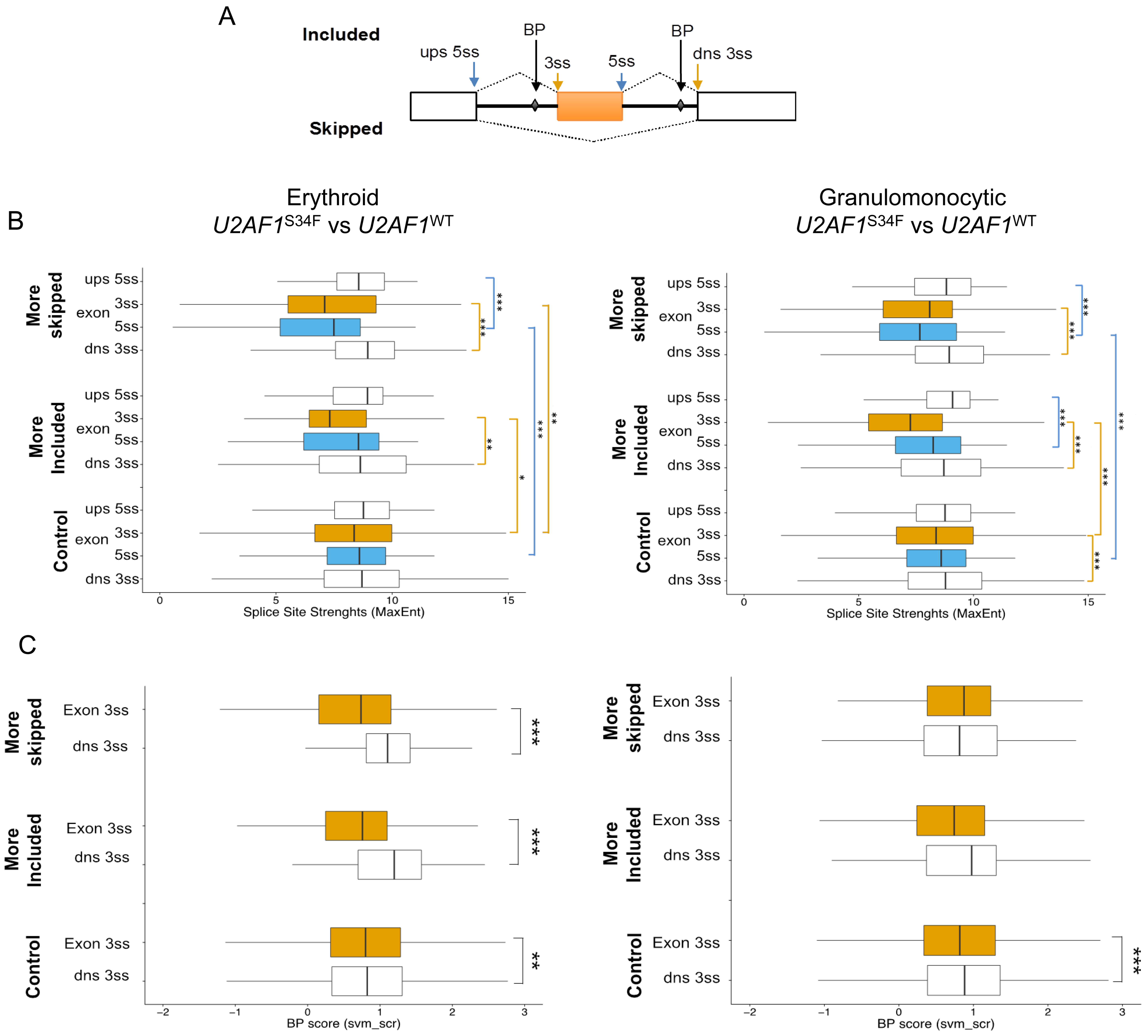

Supplemental Figure 2. Splice site strengths and BP scores for cassette exons regulated by U2AF1^{S34F}. (A) Schematic representation of cassette exons (orange) and locations of the different features analyzed. (B) Splice site strength and (C) BP scores were determined for the different data sets: Exons more Included, more Skipped upon U2AF1^{S34F} overexpression and non regulated SE control exons. For each data set, splice site scores (B) or BP scores (C) are plotted; 5'ss (blue), 3'ss (orange), upstream 5'ss (white) and downstream 3'ss (white). Boxplot's whiskers represent 1.5 IQR and outliers are not shown. Statistically significant differences (Kruskal-Wallis followed by Mann-Whitney U tests with Bonferroni correction) are marked, p-value < 0.05 (*), p-value< 0.01 (**), p-value <0.001(***), and lines are colored to show comparisons between 3'ss (orange) or 5'ss (blue).

Supplemental Figure 3

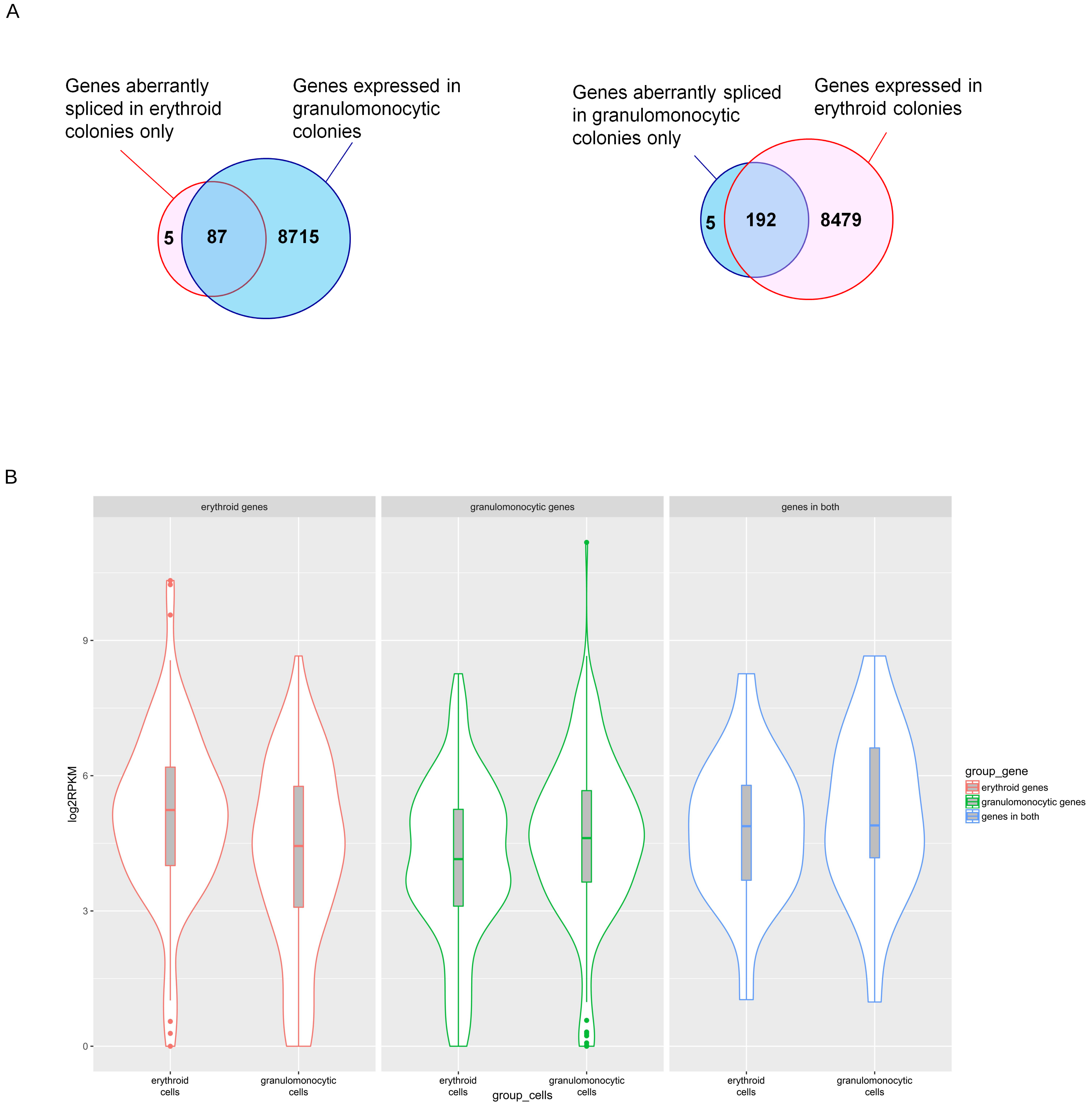

Supplemental Figure 3. **Expression levels of aberrantly spliced genes.** (A) Venn diagrams showing the overlap between genes showing aberrant splicing in erythroid colonies and genes that are expressed in granulomonocytic colonies, and between genes showing aberrant splicing in granulomonocytic colonies and genes that are expressed in erythroid colonies. The large majority of genes aberrantly spliced in either erythroid or granulomonocytic lineage only were also expressed in the other lineage. (B) Violin plots showing the distribution of the expression levels (log2rpkm) of the aberrantly spliced genes identified in our study (from Figure 3F).

Supplemental Figure 4

A

Gene	Aberrant splicing event	Affected lineage
SMARCA5	Skipping of exon 14	erythroid
ITGB3BP	Inclusion of exon 2	granulomonocytic
ATR	Inclusion of exon 47	granulomonocytic

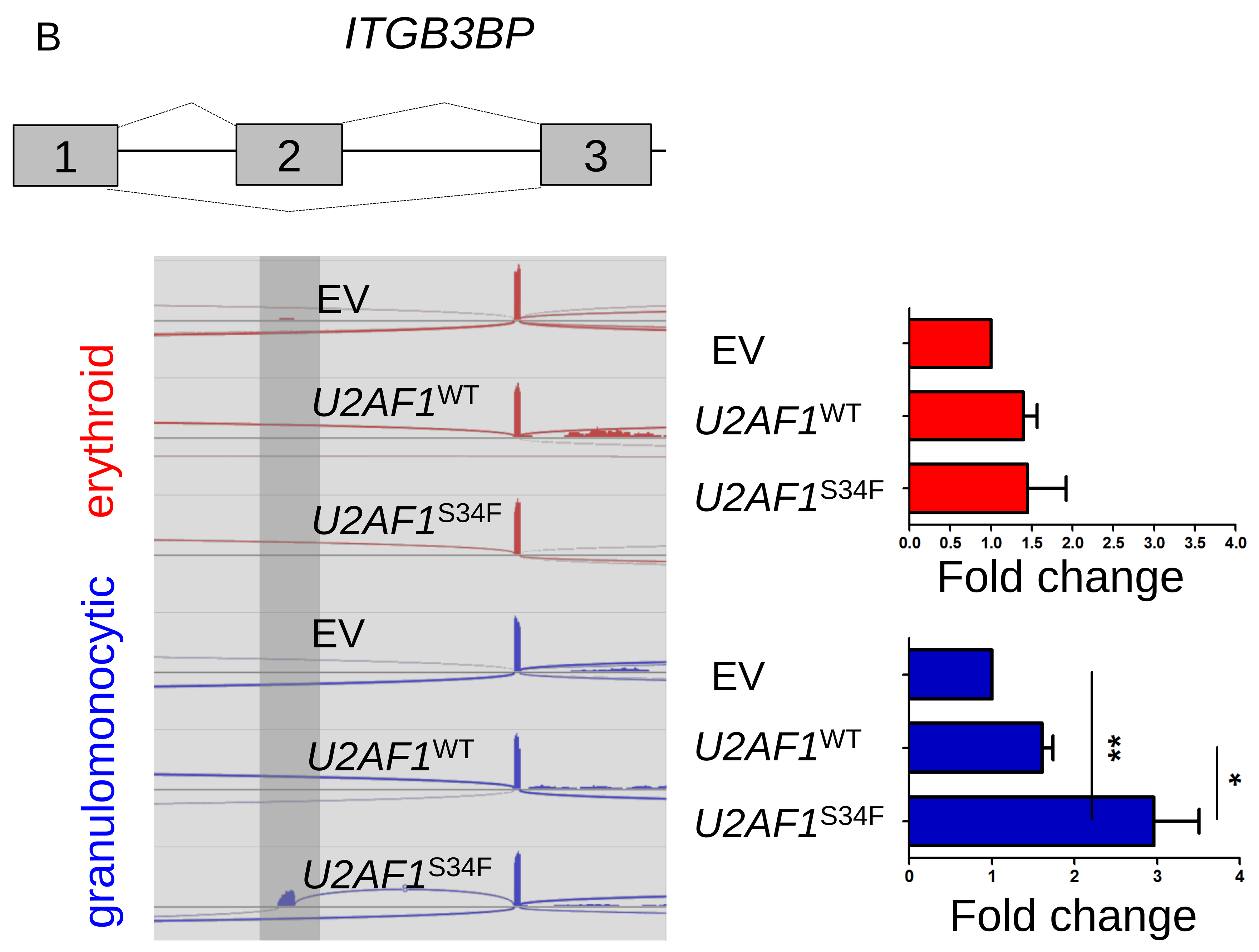

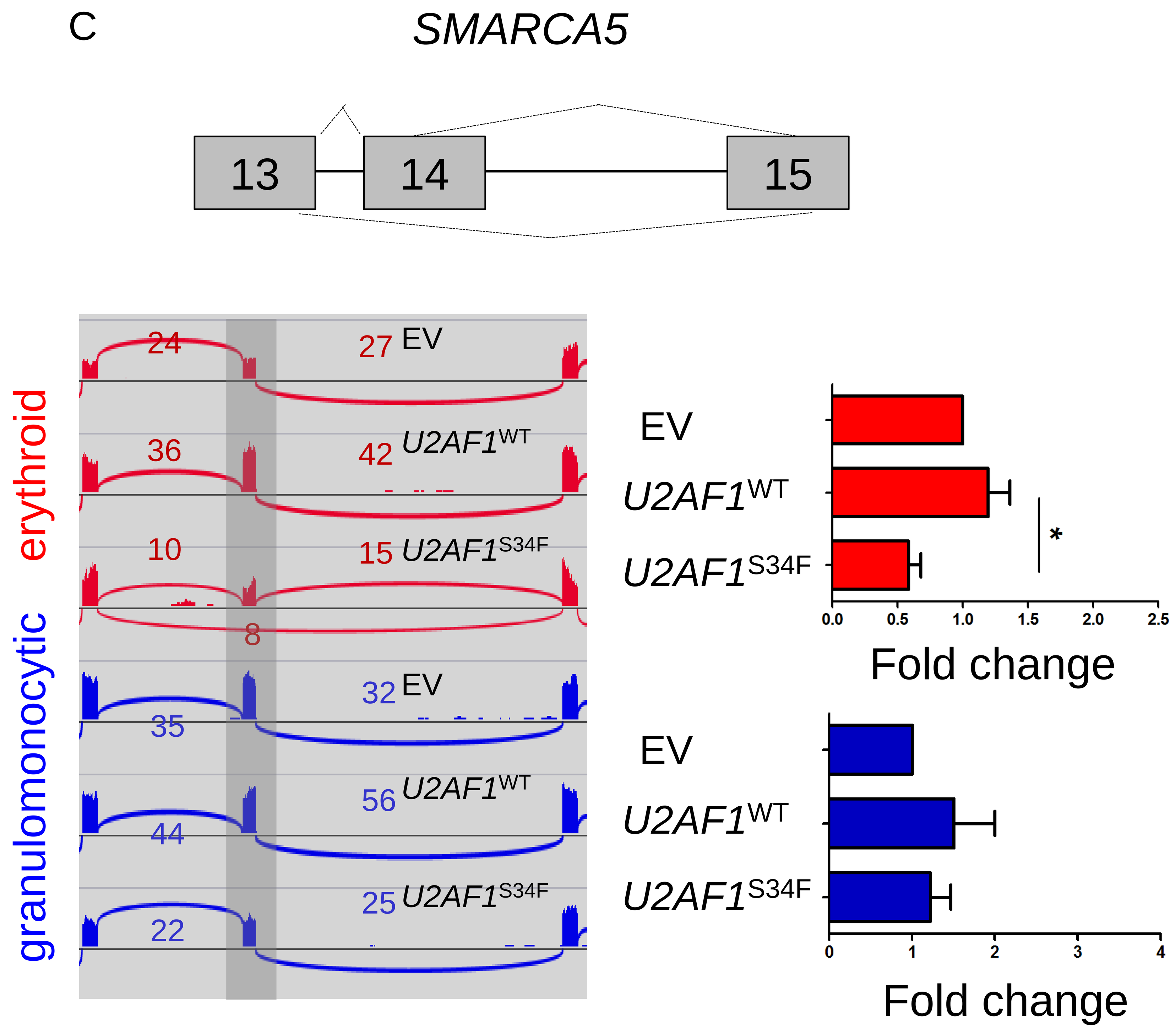

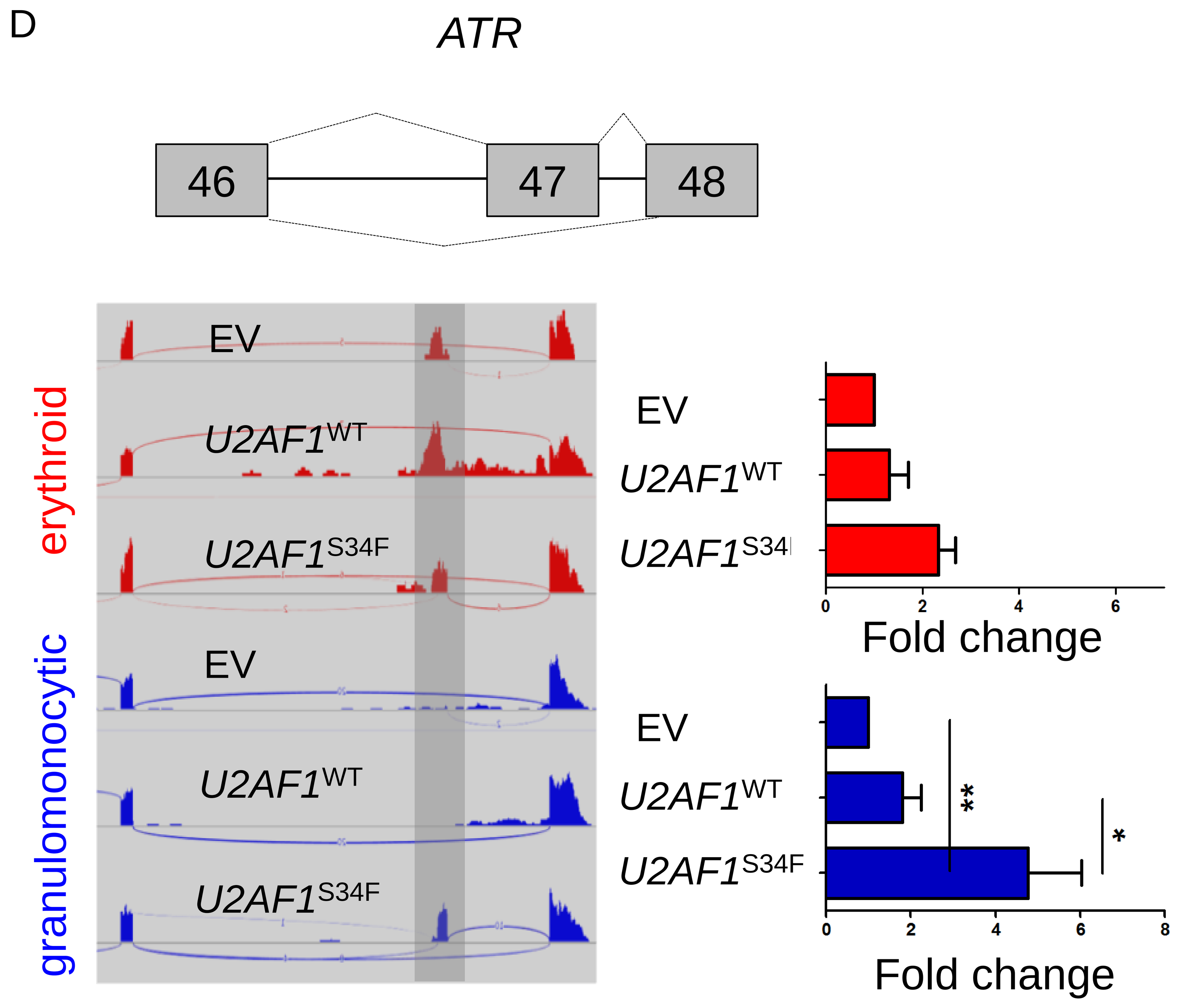

Supplemental Figure 4. Measurement of lineage-specific splicing alterations in *U2AF1*^{S34F} erythroid and granulomonocytic cells by isoform-specific qRT-PCR. (A) Genes of interest that exhibit differential aberrant splicing between *U2AF1*^{S34F} erythroid and granulomonocytic colonies (*ITGB3BP*, *SMARCA5* and *ATR*). Measurement of lineage-specific splicing alteration in (B) *ITGB3BP*, (C) *SMARCA5* and (D) *ATR*. Left panel: sashimi plots illustrating RNA sequencing results of *ITGB3BP*, *SMARCA5* and *ATR* in erythroid and granulomonocytic colonies. For each gene, only the region affected by aberrant splicing is shown and highlighted in grey. Right panels: expression of the isoform associated with aberrant splicing by *U2AF1*^{S34F} in transduced cells was measured by isoform-specific qRT-PCR relative to EV and *U2AF1*^{WT} control (red bars: erythroid cells; blue bars: granulomonocytic cells). Results in each bar graph were obtained from 5 independent experiments. Bar graphs show mean+SEM. *P<0.05, 1-way ANOVA with repeated measures using Tukey's post-test.

Supplemental Figure 5

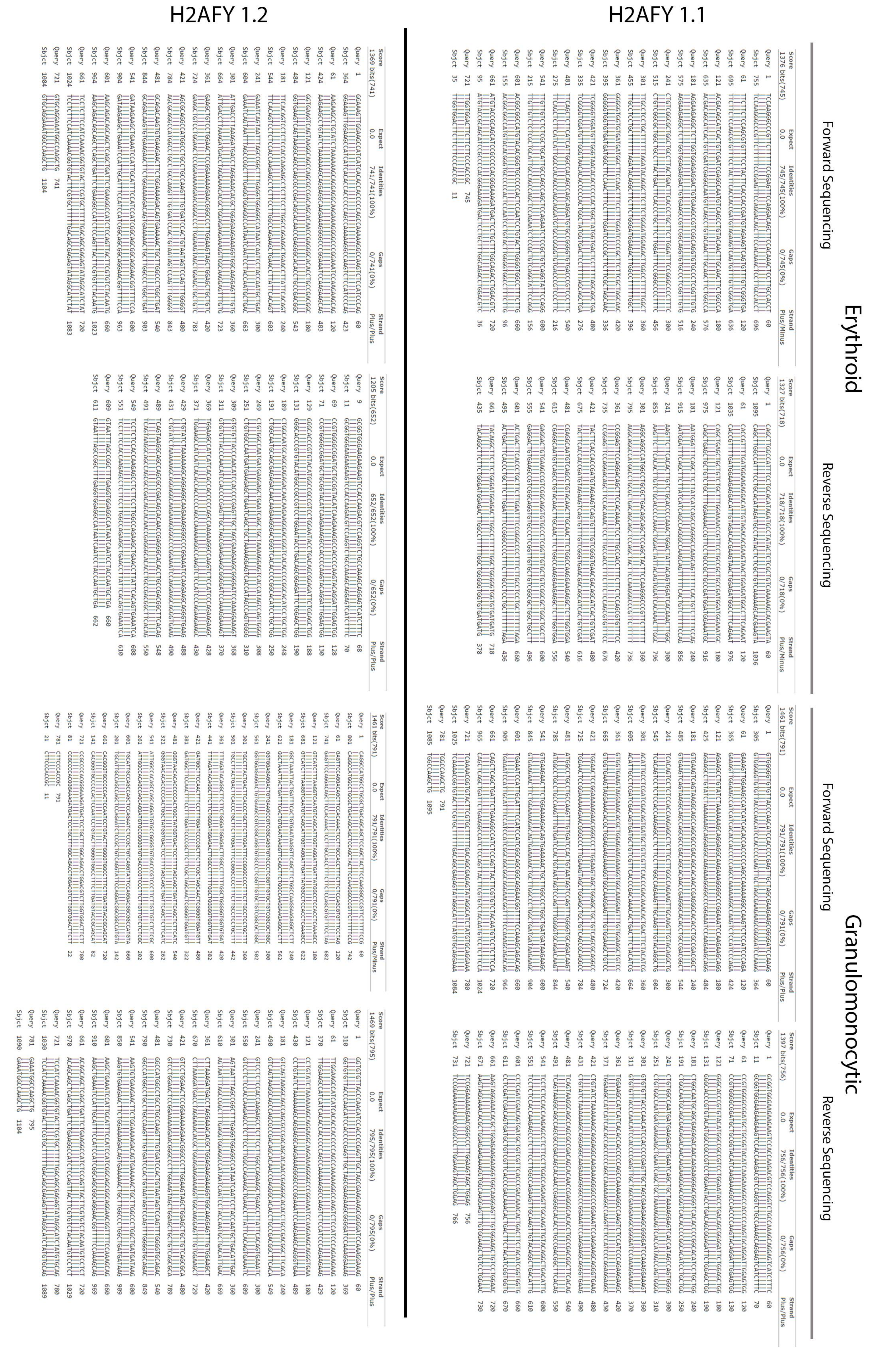

Supplemental Figure 5. Alignment of *H2AFY* isoform sequences obtained by Sanger sequencing.

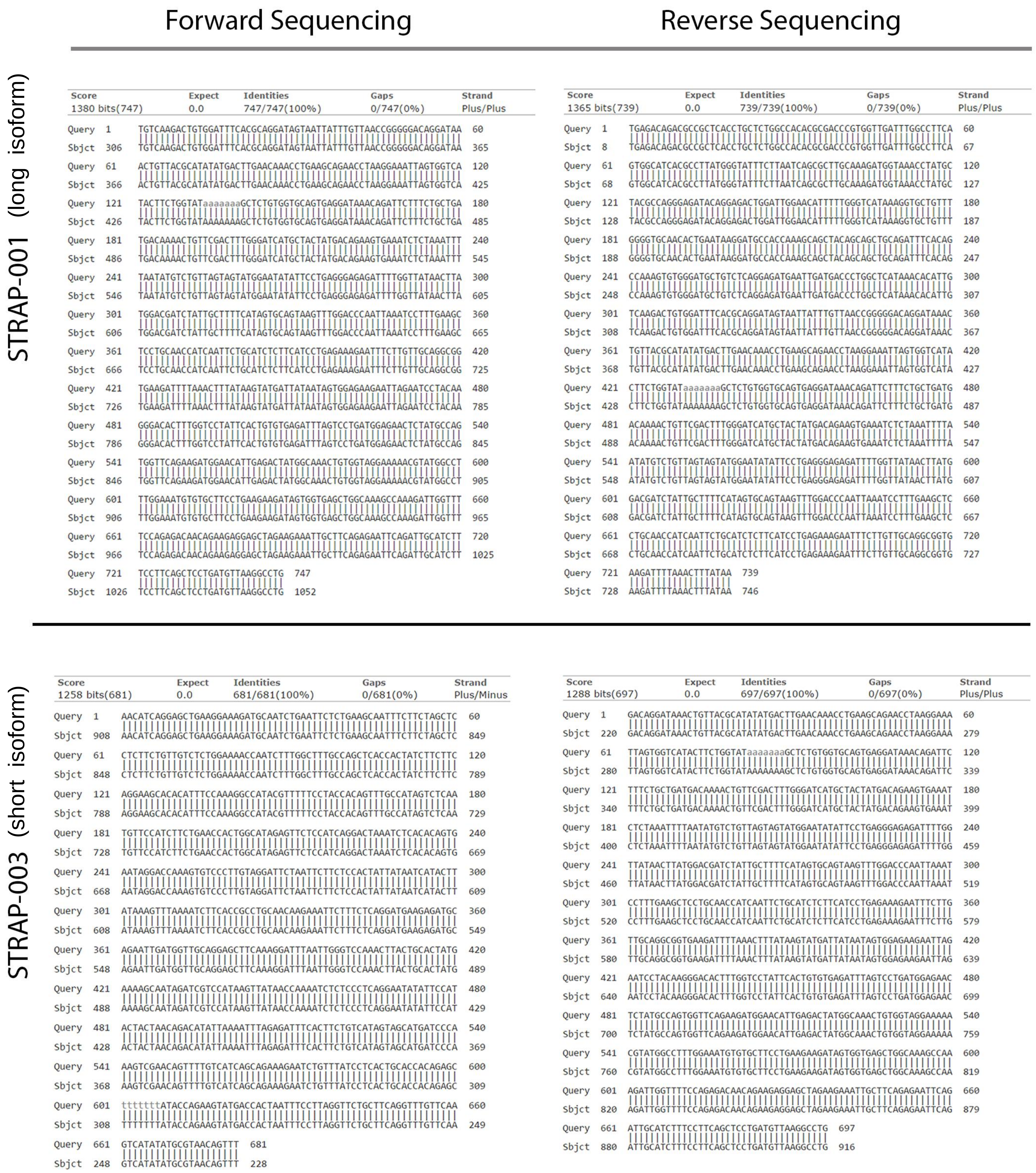

Supplemental Figure 6. Alignment of *STRAP* isoform sequences obtained by Sanger sequencing.

Supplemental Figure 7

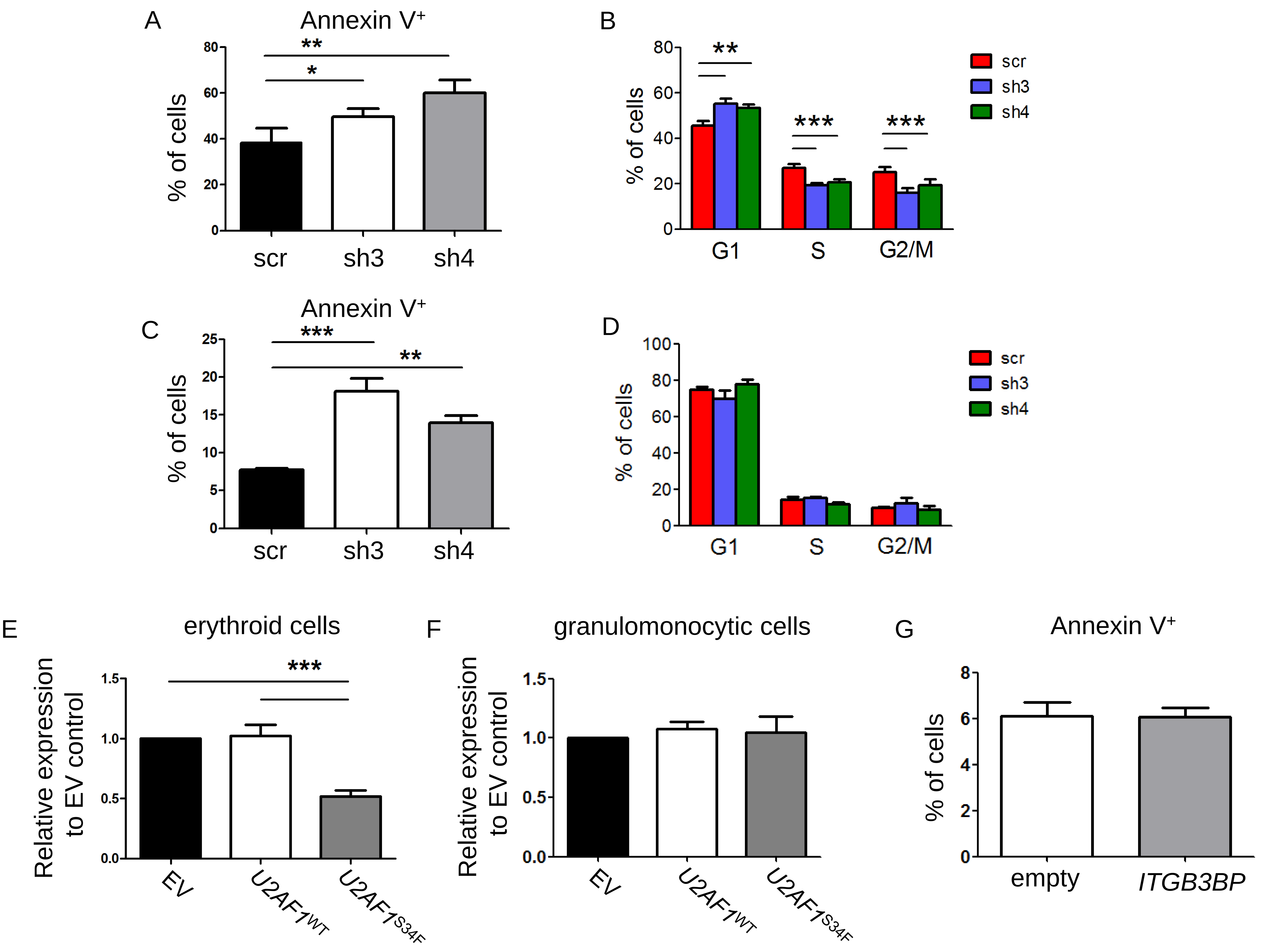

Supplemental Figure 7. Effects of *H2AFY* isoform 1.1 knockdown, *STRAP* knockdown and *ITGB3BP* overexpression in transduced hematopoietic progenitors differentiated towards the erythroid and granulomonocytic lineages. (A) Apoptosis measured by Annexin V staining and flow cytometry in transduced erythroid cells with *H2AFY* isoform 1.1 knockdown on day 11 of culture. (B) Cell cycle analysis of transduced erythroid cells with *H2AFY* isoform 1.1 knockdown on day 11 of culture. (C) Apoptosis measured by Annexin V staining and flow cytometry in transduced granulomonocytic cells with *H2AFY* isoform 1.1 knockdown on day 11 of culture. (D) Cell cycle analysis of transduced granulomonocytic cells with *H2AFY* isoform 1.1 knockdown on day 11 of culture. (E) Expression levels of *STRAP* in erythroid cells transduced with EV, *U2AF1*^{WT} or *U2AF1*^{S34F} determined using qRT-PCR. (F) Expression levels of *STRAP* in granulomonocytic cells transduced with EV, *U2AF1*^{WT} or *U2AF1*^{S34F} determined using qRT-PCR. (G) Apoptosis measured by Annexin V staining and flow cytometry in transduced granulomonocytic cells with *ITGB3BP* overexpression on day 11 of culture. Results in each bar graph in panel (A), (B), (C) and (D) were obtained from 6 independent experiments. Results in each bar graph in panel (E), (F) and (G) were obtained from 5, 5 and 6 independent experiments respectively. Bar graphs show mean+SEM. P values were calculated by 1-way ANOVA with repeated measures using Tukey's post-test. *P<0.05, **P<0.01 and ***P<0.001.

Supplemental Figure 8

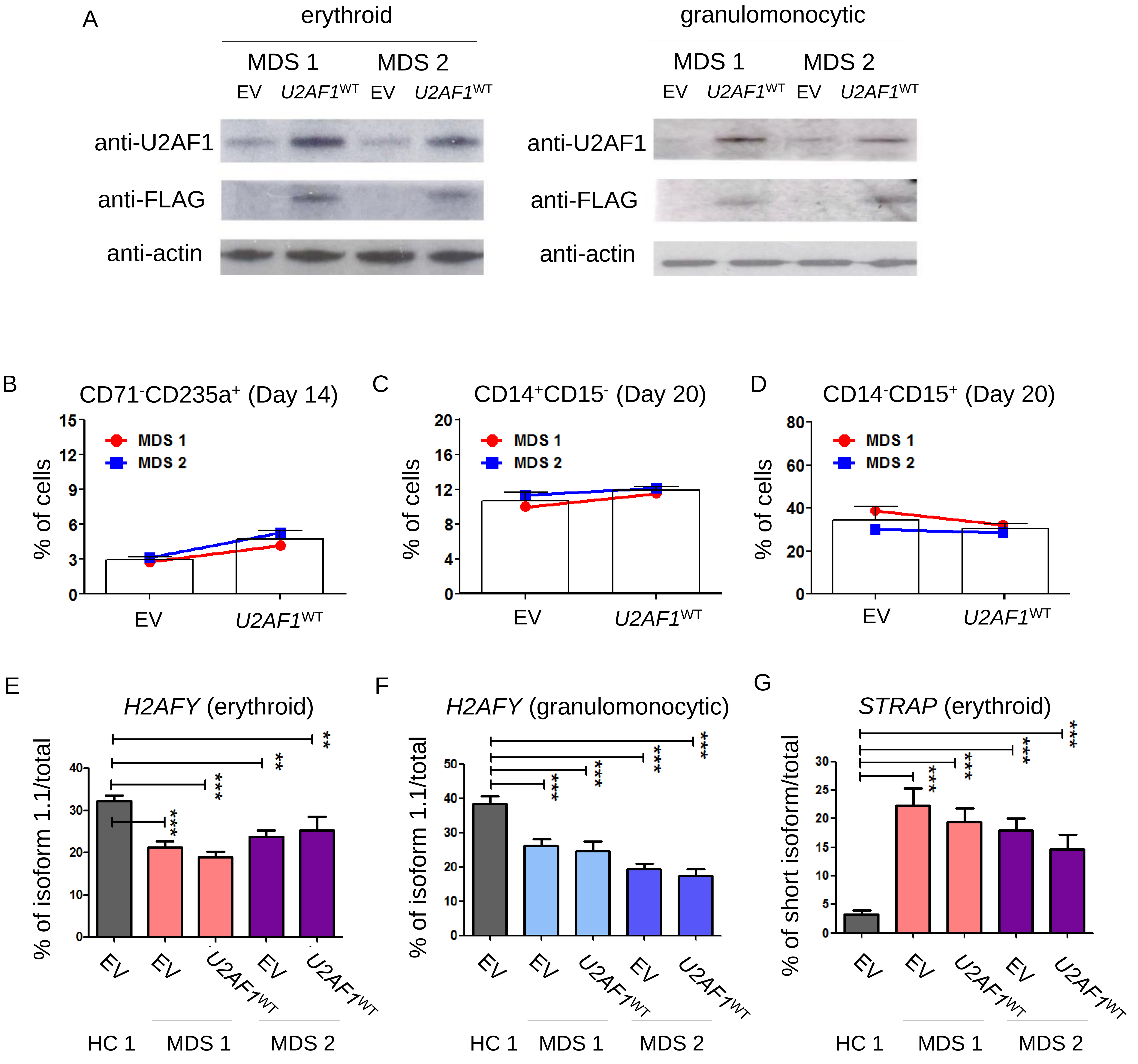

Supplemental Figure 8. Effects of U2AF1^{WT} overexpression on *U2AF1*^{S34F} MDS hematopoietic progenitors differentiated towards the erythroid and granulomonocytic lineages. (A) Expression levels of U2AF1^{WT} in *U2AF1*^{S34F} MDS erythroid and granulomonocytic cells (day 11) transduced with EV or *U2AF1*^{WT} determined using Western blotting. (B-D) Effects of U2AF1^{WT} overexpression on erythroid and granulomonocytic differentiation of *U2AF1*^{S34F} MDS hematopoietic progenitors. (B) Late erythroid (CD71^{-}CD235a^{+}) cell population on day 14 of culture, and (C) monocytic (CD14^{+}CD15^{-}) and (D) granulocytic (CD14^{-}CD15^{+}) cell populations on day 20 of culture were measured by flow cytometry. (E-F) Ratio of *H2AFY* isoform 1.1 in EV or *U2AF1*^{WT} transduced (E) erythroid cells (Day 14) and (F) granulomonocytic cells (Day 20) in culture measured by RT-PCR and gel electrophoresis. (G) Ratio of *STRAP* short isoform in EV or *U2AF1*^{WT} transduced erythroid cells (Day 14) in culture was measured by RT-PCR and gel electrophoresis. In panel (E-G), quantification of altered splicing events in gel was performed by ImageJ. Results in each bar graph were obtained from 3 independent experiments in panels (E-G). Results are shown as mean ± SEM. P values in panels E-G were calculated by 1-way ANOVA using Tukey's post-test. *P<0.05, **P<0.01 and ***P<0.001.

Supplemental Table 1

Sequence of primers used in this study.

**Name**	**Sequence (5'>3')**	**Application**
U2AF1 S34F F	ATGGCGGAGTATCTGGCCTC	Sequencing of *U2AF1*^{S34F} mutation
U2AF1 S34F R	TCAGAATCGCCCAGATCTTT	Sequencing of *U2AF1*^{S34F} mutation
H2AFY shRNA 3	TCGACAGTGATGCTGTCGT	Knockdown *H2AFY* isoform 1.1
H2AFY shRNA 4	GTCGTTCACCCGACAAACA	Knockdown *H2AFY* isoform 1.1
H2AFY isoform1.1 F	CAGGGTGAAGTCAGTAAGGC	Isoform-specific qRT-PCR and RT-PCR for *H2AFY* isoform1.1 and total
H2AFY isoform1.1 R	CTTCACCACCGATGTAGAAG	Isoform-specific qRT-PCR and RT-PCR for *H2AFY* isoform1.1 and total
H2AFY isoform1.2 F	CTTTGAGGTGGAGGCCATAA	Isoform-specific qRT-PCR for *H2AFY* isoform1.2
H2AFY isoform1.2 R	CTACTTCCAAGGGCCCGTTC	Isoform-specific qRT-PCR RT-PCR for *H2AFY* isoform1.2 and total
STRAP isoform F	CCTATGCTACGCCAGGGAGATAC	Isoform-specific qRT-PCR for *STRAP*
STRAP isoform R	CTGCGTGAAATCCACAGTCTTGAC	Isoform-specific qRT-PCR for *STRAP*
STRAP ex8 qRT F	CCTACAAGGGCAACTTTGGTCCTA	qRT-PCR for *STRAP*
STRAP ex9 qRT R	CTAGCTCCTCTTCTGTTGTCTCTGG	qRT-PCR for *STRAP*
STRAP ex1 RT F	AATGAGACAGACGCCGCTCA	RT-PCR for *STRAP* long and short isoform
STRAP ex3 RT R	CTGCGTGAAATCCACAGTCTTGAC	RT-PCR for *STRAP* long and short isoform
SMARCA5 F	GAGTACTGCAGGTTGGATGGTCAG	Isoform-specific qRT-PCR for *SMARCA5*
SMARCA5 R	ACACTCTGACTGTCTTAGTCTGCCC	Isoform-specific qRT-PCR for *SMARCA5*
ITGB3BP F	CCGTTCACTGCAACATCTGCT	Isoform-specific qRT-PCR for *ITGB3BP*
ITGB3BP R	GCTCTTCAGAACTTGTGGGAGA	Isoform-specific qRT-PCR for *ITGB3BP*
ATR F	TGGAATGGGTCCTATGGGAACAGAGGGTCT	Isoform-specific qRT-PCR for *ATR*
ATR R	GTTCATCAGGATCCTTGTGAGGC	Isoform-specific qRT-PCR for *ATR*

Supplemental Table 1

Continued

**Name**	**Sequence (5'>3')**	**Application**
H2AFY Cloning F	GCGGTGGGAAGAAGAAGTCCAC	Cloning of H2AFY to confirm full length isoform expression
H2AFY Cloning R	CAGCTTGGCCATTTCCTGCAC	Cloning of H2AFY to confirm full length isoform expression
STRAP Cloning F	TGAGACAGACGCCGCTCACCT	Cloning of STRAP to confirm full length isoform expression
STRAP Cloning R	CAGGCCTTAACATCAGGAGCTGA	Cloning of STRAP to confirm full length isoform expression
h*ATG7*_proximal CP F	GCTGCTGAGATCTGGGACAT	SYBR green qRT-PCR assessment of ATG7 proximal polyadenylation site usage
h*ATG7*_proximal CP R	CAGAGGGGGGAATCCCA	SYBR green qRT-PCR assessment of ATG7 proximal polyadenylation site usage
h*ATG7*_distal CP F	GGGCATCGTCTTTCCTGCTA	SYBR green qRT-PCR assessment of ATG7 distal polyadenylation site usage
h*ATG7*_distal CP R	TGGCTACTTTGGGAGAAGCG	SYBR green qRT-PCR assessment of ATG7 distal polyadenylation site usage
U2AF1 pyro F	TTCAAAATTGGAGCATGTCG	U2AF1 S34 Pyrosequencing assay
U2AF1 pyro R	Biotin- ATGGTCTGGCTAAACGTCG	U2AF1 S34 Pyrosequencing assay
U2AF1 pyro seq	AATTGGAGCATGTCGTC	U2AF1 S34 Pyrosequencing assay

Supplemental Table 2. RNA-seq quality control metrics.

Sample ID	Sample name	Lane	Total reads	Total mapped reads	Uniquely mapped number	Intragenic Rate	Intronic Rate	Exonic Rate	Intergenic Rate	Expression Profiling Efficiency	Split Reads	Transcripts Detected	Genes Detected	Mean Per Base Cov.	Mean CV
WTCHG_165332_258	erythroid-empty-1	one lane	25546477	18616536	13535386	0.911	0.239	0.672	0.089	0.6724	6508934	24821	12737	16.79	0.935
WTCHG_171117_277	erythroid-empty-2	lane1	15065510	13093191	8799374	0.912	0.203	0.708	0.088	0.7084	5790416	24073	12262	13.09	0.758
WTCHG_172885_277	erythroid-empty-2	lane2	13372436	11592765	7790067	0.912	0.202	0.709	0.088	0.7093	5123537	23790	12095	11.46	0.773
WTCHG_171117_278	erythroid-empty-3	lane1	17861947	15460116	13198309	0.858	0.233	0.625	0.142	0.6246	5775406	26045	13377	19.55	0.734
WTCHG_172885_278	erythroid-empty-3	lane2	15959058	13798535	11784152	0.857	0.232	0.625	0.142	0.6250	5160843	25872	13268	17.29	0.749
WTCHG_165332_260	erythroid-s34f-1	one lane	27811937	22175539	19260331	0.871	0.248	0.623	0.128	0.6232	8450704	26094	13407	30.32	0.730
WTCHG_171117_257	erythroid-s34f-2	lane1	18712942	16333021	12527116	0.878	0.176	0.702	0.122	0.7021	7175933	25724	13195	20.45	0.716
WTCHG_172885_257	erythroid-s34f-2	lane2	15811020	13783768	10566838	0.877	0.175	0.702	0.123	0.7021	6061265	25416	12997	17.40	0.720
WTCHG_171117_258	erythroid-s34f-3	lane1	14814430	12767234	8009230	0.934	0.175	0.759	0.066	0.7591	5751814	23418	11876	11.48	0.777
WTCHG_172885_258	erythroid-s34f-3	lane2	15067887	12979509	8148055	0.934	0.174	0.760	0.066	0.7598	5848850	23489	11884	11.72	0.766
WTCHG_165332_259	erythroid-wt-1	one lane	27279491	21159579	16365090	0.882	0.253	0.628	0.118	0.6284	6938049	25666	13237	22.58	0.919
WTCHG_171117_279	erythroid-wt-2	lane1	15471495	13766562	8681376	0.936	0.177	0.759	0.064	0.7591	6501764	23754	12111	13.99	0.767
WTCHG_172885_279	erythroid-wt-2	lane2	14636258	13017120	8181956	0.936	0.176	0.760	0.063	0.7602	6158388	23548	12020	13.17	0.776
WTCHG_171117_280	erythroid-wt-3	lane1	16170639	14000232	8840547	0.916	0.181	0.735	0.084	0.7352	6361189	24313	12363	13.91	0.744
WTCHG_172885_280	erythroid-wt-3	lane2	15049777	13012166	8180752	0.916	0.180	0.737	0.083	0.7366	5918893	24209	12314	12.87	0.755
WTCHG_165332_261	granulomonocytic-empty-1	one lane	27820205	23194583	22442598	0.884	0.188	0.697	0.115	0.6967	9759322	26139	13462	34.91	0.802
WTCHG_171117_259	granulomonocytic-empty-2	lane1	15241845	13616677	13057942	0.872	0.246	0.626	0.127	0.6261	5320919	25048	12834	20.05	0.730
WTCHG_172885_259	granulomonocytic-empty-2	lane2	16364139	14611988	14019233	0.873	0.246	0.627	0.127	0.6266	5710968	25186	12916	21.59	0.742
WTCHG_171117_260	granulomonocytic-empty-3	lane1	18517334	16535205	15936610	0.890	0.202	0.688	0.110	0.6884	7178954	25633	13123	25.15	0.771
WTCHG_172885_260	granulomonocytic-empty-3	lane2	16500313	14733089	14211779	0.890	0.201	0.689	0.110	0.6890	6409513	25467	13048	22.48	0.773
WTCHG_165332_263	granulomonocytic-s34f-1	one lane	29075241	24749666	23945787	0.889	0.218	0.671	0.111	0.6709	10011758	26437	13617	35.78	0.770
WTCHG_171117_263	granulomonocytic-s34f-2	lane1	13525385	11544333	11020753	0.846	0.236	0.610	0.153	0.6103	4190498	23813	12198	17.59	0.831
WTCHG_172885_263	granulomonocytic-s34f-2	lane2	14143648	12069933	11533003	0.846	0.236	0.611	0.153	0.6106	4381522	23834	12197	18.45	0.815
WTCHG_171117_264	granulomonocytic-s34f-3	lane1	15738365	13336318	12758067	0.838	0.271	0.567	0.162	0.5671	4414612	24711	12693	18.55	0.803
WTCHG_172885_264	granulomonocytic-s34f-3	lane2	14343654	12141383	11627026	0.837	0.270	0.567	0.163	0.5673	4027735	24607	12639	16.76	0.784
WTCHG_165332_262	granulomonocytic-wt-1	one lane	28075831	22967423	22256097	0.872	0.164	0.707	0.128	0.7073	9983910	24096	12362	33.07	0.782
WTCHG_171117_261	granulomonocytic-wt-2	lane1	13504521	11713391	11157958	0.850	0.294	0.556	0.149	0.5563	3982596	24054	12323	15.93	0.791
WTCHG_172885_261	granulomonocytic-wt-2	lane2	14511454	12577442	11990629	0.851	0.294	0.556	0.149	0.5563	4277559	24190	12369	17.09	0.813
WTCHG_171117_262	granulomonocytic-wt-3	lane1	15999726	14136893	13490820	0.880	0.184	0.696	0.120	0.6963	6127524	25572	13071	20.82	0.797
WTCHG_172885_262	granulomonocytic-wt-3	lane2	17118242	15115345	14438060	0.880	0.184	0.697	0.119	0.6968	6556334	25623	13132	22.24	0.780
